# Synthesis of higher carboxylic acids from ethers, CO_2_ and H_2_

**DOI:** 10.1038/s41467-019-13463-0

**Published:** 2019-12-04

**Authors:** Ying Wang, Qingli Qian, Jingjing Zhang, Bernard Baffour Asare Bediako, Zhenpeng Wang, Huizhen Liu, Buxing Han

**Affiliations:** 1 Beijing National Laboratory for Molecular Sciences, CAS Key Laboratory of Colloid, Interface and Chemical Thermodynamics, CAS Research/Education Center for Excellence in Molecular Sciences, Institute of Chemistry, Chinese Academy of Sciences, Beijing, 100190 China; 2 School of Chemistry and Chemical Engineering, University of Chinese Academy of Sciences, Beijing, 100049 P. R. China; 3 Physical Science Laboratory, Huairou National Comprehensive Science Center, No. 5 Yanqi East Second Street, Beijing, 101400 China; 4 Shanghai Key Laboratory of Green Chemistry and Chemical Processes, School of Chemistry and Molecular Engineering, East China Normal University, Shanghai, 200062 China

**Keywords:** Homogeneous catalysis, Synthetic chemistry methodology

## Abstract

Synthesis of higher carboxylic acids using CO_2_ and H_2_ is of great importance, because CO_2_ is an attractive renewable C1 resource and H_2_ is a cheap and clean reductant. Herein we report a route to produce higher carboxylic acids via reaction of ethers with CO_2_ and H_2_. We show that the reaction can be efficiently catalyzed by an IrI_4_ catalyst with LiI as promoter at 170 °C, 5 MPa of CO_2_ and 2 MPa of H_2_. The catalytic system applies to various ether substrates. The mechanistic study indicates that the ethers are converted to olefins, which are further transformed into alkyl iodides. The higher carboxylic acids are produced by carbonylation of alkyl iodides with CO generated in situ via RWGS reaction. This report offers an alternative strategy of higher carboxylic acid synthesis and CO_2_ transformation.

## Abstract (web summary)

CO_2_ conversion into value-added chemicals is a promising strategy to exploit renewable feedstocks. Here, the authors report the iridium-catalysed synthesis of higher carboxylic acids from ethers, CO_2_ and H_2_.

## Introduction

As an attractive C1 resource, CO_2_ can be converted to various kinds of value-added products^1–5^. Carboxylic acids are an important class of chemicals. Synthesis of carboxylic acids using CO_2_ as a C1 synthon is an interesting topic. In the past decades, great advances have been made in synthesis of formic acid via CO_2_ hydrogenation^6–10^. Synthesis of higher carboxylic acids using CO_2_ has also been extensively studied^11–17^. However, at present stage expensive or air/water sensitive substrates, such as Grignard and other organometallic reagents, unsaturated hydrocarbons and/or organic halides, were usually used in stoichiometric reactions. Moreover, silanes and/or metallic reducing agents were often required to produce higher carboxylic acids. Hydrogen gas is a clean, cheap and easily available reductant. Undoubtedly, synthesis of higher carboxylic acids using CO_2_ and H_2_ is of great importance. In a pioneering work, higher carboxylic acids were successfully synthesized by reaction of olefins, CO_2_ and H_2_ using four-component catalytic system, containing {RhCl(CO)_2_}_2_ catalyst, PPh_3_ ligand, CH_3_I promoter, and acidic additive (p-TsOH·H_2_O)^18^. Later, it was reported that acetic acid (AcOH) could be produced via methanol hydrocarboxylation with CO_2_ and H_2_ at above 180 °C, which was accelerated by Ru-Rh bimetallic catalyst, imidazole ligand and LiI promoter^19^. This reaction could also be efficiently accelerated by Rh catalyst, in the presence of 4-methylimidazole ligand, LiCl and LiI promoters^20^. Therefore, in the previous reports of higher carboxylic acid synthesis using CO_2_ and H_2_, Rh-based four catalytic components were used to accelerate the reaction.

**Fig. 1:**
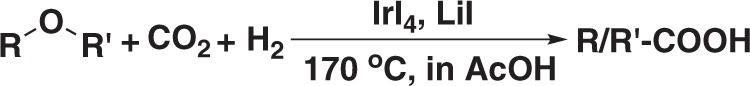
Synthesis of higher carboxylic acids via reaction of ether with CO_2_ and H_2_. Higher carboxylic acids can be efficiently produced from ether, CO_2_ and H_2_, where a simple Ir catalyst was utilized.

Ethers are cheap and basic chemicals that can be obtained from various sources including biomass, but they have not been utilized as substrate in producing higher carboxylic acids with CO_2_. In general, ethers are less reactive than olefins and/or alcohols. Herein we report a strategy to produce higher carboxylic acids from ethers, CO_2_ and H_2_. The reaction can be effectively accelerated by a simple catalytic system consisting of IrI_4_ catalyst and LiI promoter at 170 °C (Fig. 1). The catalytic system has good substrate adaptability and various ethers could be converted to higher carboxylic acids. This route is of practical importance because some of the higher carboxylic acids are much more valuable than the corresponding ether substrate (Supplementary Table 1). In addition, the price of Ir is much lower than that of Rh although both are known as noble metals.

## Results and Discussion

### Catalytic system

Tetrahydrofuran (THF) is a bulk chemical, which can be obtained from various feedstocks including biomass^21^. In addition, the C_5_ carboxylic acids are much more expensive than THF. Therefore, we adopted THF as model ether to study the catalytic system (Table 1). The reaction could be efficiently accelerated by IrI_4_ catalyst and LiI promoter in AcOH solvent at 170 °C, and the yield of C_5_ carboxylic acids reached 70% after 16 h (entry 1). The products contained two isomers, i.e., pentanoic acid and 2-methylbutanoic acid, and their molar ratio was 58:42. A little C_6_ carboxylic acids were also formed in the reaction. The rest of the THF substrate was converted to butane. In addition, trace of methane was also detected. We also tried different Ir catalyst precursors, such as Ir(CO)(PPh_3_)_2_Cl, Ir(CO)_2_(acac), and IrCl_3_, the results indicated that they were not as efficient as IrI_4_ (Supplementary Table 2). We set the reaction time at 8 h and tested other catalytic systems. The IrI_4_ catalyst was essential to the reaction because no target product was observed without it (entries 2–3). The Rh catalyst was effective for synthesis of carboxylic acids via olefin and/or alcohol hydrocarboxylation with CO_2_ and H_2_^18–20^. Whereas in this work no product was obtained when RhI_3_ was used to replace IrI_4_ (entry 4). We also tried other metal compounds, such as CoCl_2_, FeI_2_, RuI_3_, PdCl_2_, and PtCl_2_, but the results were poor (entries 5–9). Hence IrI_4_ was a suitable catalyst for this reaction.

**Table 1:** Different catalytic systems for synthesizing C_5_ carboxylic acids from THF, CO_2_, and H_2_.


Entry	Catalyst precursor	Promoter	Solvent	Yield of (2a + 2a’) [%]^a^
1^b^	IrI_4_	LiI	AcOH	70
2	IrI_4_	LiI	AcOH	44
3	–	LiI	AcOH	0
4	RhI_3_	LiI	AcOH	<1
5	CoCl_2_	LiI	AcOH	<1
6	FeI_2_	LiI	AcOH	0
7	RuI_3_	LiI	AcOH	<1
8	PdCl_2_	LiI	AcOH	<1
9	PtCl_2_	LiI	AcOH	<1
10	IrI_4_	–	AcOH	0
11	IrI_4_	NaI	AcOH	2
12	IrI_4_	KI	AcOH	<1
13	IrI_4_	MgI_2_	AcOH	0
14	IrI_4_	ZnI_2_	AcOH	0
15	IrI_4_	LiCl	AcOH	<1
16	IrI_4_	LiBr	AcOH	<1
17	IrI_4_	I_2_	AcOH	10
18	IrI_4_	CH_3_I	AcOH	<1
19	IrI_4_	LiI	hexanoic acid	37
20	IrI_4_	LiI	DMF	0
21	IrI_4_	LiI	Toluene	0
22	IrI_4_	LiI	DMSO	0
23	IrI_4_	LiI	H_2_O	0
24	IrI_4_	LiI	1-ethyl-3-methylimidazolium acetate	0

Reaction conditions: 20 μmol catalyst precursor, 2 mmol promoter, 0.6 mL solvent, 2.45 mmol THF, 5 MPa CO_2_ (68 mmol) and 2 MPa H_2_ (at room temperature), 170 °C, 8 h

^a^Yield is based on THF feedstock (100 × moles of 2a and 2a’ per mole of THF feedstock)

^b^The reaction time was 16 h and the 2a accounted for 58% of total products (2a + 2a’)

The promoter was also crucial to the reaction. Without LiI, the target reaction did not occur (entry 10). When other alkali metal iodides (NaI, KI) were utilized in the reaction, the yields were much lower than that using LiI (entries 2, 11, 12). Moreover, their promoting effects followed the order: LiI > NaI > KI. We also tested alkaline-earth metal iodide (MgI_2_) or transition metal iodide (ZnI_2_), but the C_5_ carboxylic acids were not observed (entries 13, 14). The superiority of Li^+^ may lie in its stronger Lewis acidity and small size, which could help to activate the substrate^19,20^. When LiCl and LiBr were adopted instead of LiI, the products were hardly detectable (entries 15, 16). We further tried iodine (I_2_) and organic iodide (CH_3_I), but the results were not satisfactory (entries 17, 18). The iodide effects in organic reactions have been summarized elsewhere^22^.

The solvent effect was also studied in this work. When the reaction was conducted in hexanoic acid, instead of AcOH, the reaction took place but the yield was not as high as that in AcOH (entries 2, 19). When other common organic solvents such as DMF, toluene and DMSO were used, the reaction did not occur (entries 20–22). The reaction could not proceed in water either (entry 23). We also tried an ionic liquid, i.e., 1-ethyl-_3_-methylimidazolium acetate, whereas no target product was found after the reaction (entry 24). These suggested that the solvent acidity was crucial to the reaction. Further experiments indicated that organic acids such as AcOH, CF_3_COOH, or CH_3_SO_3_H could accelerate the reaction and their effects were similar (Supplementary Table 3), while the reaction did not take place when an inorganic Bronsted acid (H_3_PO_4_) or a Lewis acid (Al(OTf)_3_) was used. This resulted mainly from the better solubility of the reactive components in organic acids. As a cheap, safer and easily available organic acid, acetic acid was the suitable choice of the reaction solvent. In brief, the catalytic system consisting of IrI_4_, LiI, and AcOH was suitable for the reaction.

### Impact of reaction conditions

**Fig. 2:**
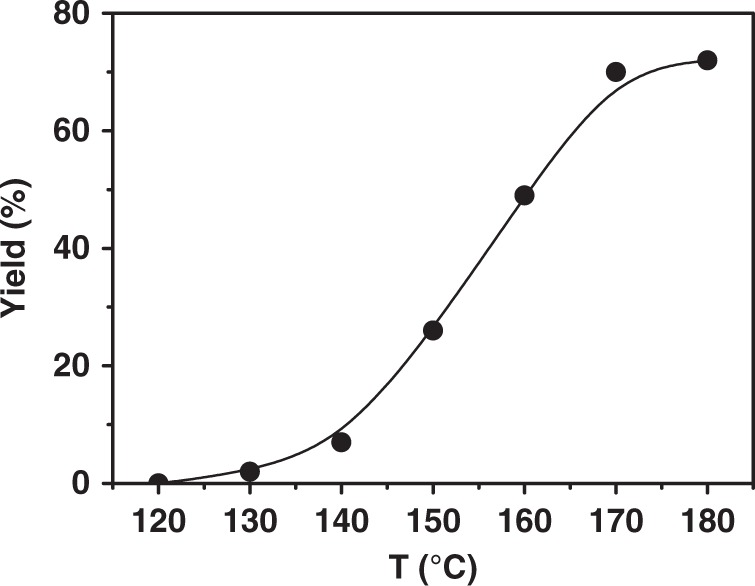
Impact of reaction temperature. Reaction conditions: 20 μmol IrI_4_, 2 mmol LiI, 0.6 mL AcOH, 2.45 mmol THF, 5 MPa CO_2_ (68 mmol) and 2 MPa H_2_ (at room temperature), 16 h.

Based on the above catalytic system, we studied the impact of reaction temperature (Fig. 2). The C_5_ carboxylic acids appeared at 130 °C, and the yield rose quickly with elevating temperature. At 170 °C, the yield of C_5_ carboxylic acids reached 70% and the increase became minor when the temperature was further raised. Consequently, the suitable reaction temperature was 170 °C. At this temperature, we investigated the effect of other reaction conditions, such as dosages of IrI_4_ and LiI, pressures of CO_2_ and H_2_. The results are shown in Supplementary Table 4. The yield of C_5_ carboxylic acids increased with the elevating IrI_4_ dosage, and the yield reached 70% when 20 μmol IrI_4_ was used in the reaction (entries 1, 2). Whereas the catalytic performance decreased slightly if the IrI_4_ dosage was further raised (entries 3, 4). The dosage of LiI also affected the reaction and the trend was similar to that of IrI_4_ (entries 2, 5–7). In addition, the suitable amount of LiI was 2 mmol. The reactant gases were needed in the reaction. Without CO_2_ and/or H_2_, no product was observed (entries 8–10). The ratio of CO_2_ and H_2_ influenced the reaction. We fixed the total pressure at 7 MPa and the best performance was obtained at 5 MPa CO_2_ and 2 MPa H_2_ (entries 2, 11–13). We further fixed the CO_2_/H_2_ ratio at 5/2 and changed the total pressure. It was found that the yield increased with the elevating total pressure, but the increase became minor when the pressure was high enough (entries 2, 14, 15). In short, the optimized reaction condition was 20 μmol IrI_4_, 2 mmol LiI, 0.6 mL AcOH, 2.45 mmol THF, 5 MPa CO_2_ (68 mmol) and 2 MPa H_2_ (at room temperature), 170 °C.

### Substrate adaptability

Based on above condition, we tested various ether substrates (Table 2). The representative GC-MS spectra are given in Supplementary Figs. 1–7. In addition, all the LC-MS graphs of the product were provided in Supplementary Figs. 8–33, the detailed peak information of which was supplied in Supplementary Tables 10–35. When the THF with one or two alkyl substituents were tried, the reaction took place but the yield of products was lower (entries 2, 3). We also tested furan and found that the reaction products and their distribution were similar to those from THF (entries 4). This suggested that furan was in situ reduced to THF before carboxylic acids were formed. After screening the cyclic ethers of different carbon chain length (C_3_, C_4_, C_5_, C_6_), we observed that they could be effectively transformed into corresponding carboxylic acids with one more C atom (entries 1, 5–7). Interestingly, besides C_4_ products, minor C_5_ carboxylic acids and trace C_6_ carboxylic acids were also formed when 1,3-epoxypropane (C_3_) was used as substrate (entry 5). Similar phenomena were also observed when other substrates with shorter alkyl chain were utilized. When olefin oxides, i.e., propylene oxide, cyclopentene oxide and cyclohexene oxide, were used as substrate respectively, the reaction proceeded well (entries 8–10). We also tried dioxane and the result showed that C_3_ and C_4_ carboxylic acids were produced (entry 11). Besides these ethers of cyclic structure, various linear alkyl ethers were transformed into corresponding carboxylic acids (entries 12–19). The ethers involved different kinds of alkyl groups, such as linear/branched alkyls, primary/secondary alkyls, and/or cycloalkyl. From these catalytic results, we can deduce that both alkyl groups in each ether participated in forming carboxylic acids. The catalytic system could also apply to various aryl alkyl ethers (entries 20–26). The alkyl group could be transformed into corresponding carboxylic acids, while the aryl group was converted to phenol and/or phenyl acetate. The presence of substituents on the benzene ring affected the yield of the carboxylic acids. The yield of the carboxylic acids slightly increased in the presence of an electron withdrawing group, while it decreased remarkably when an electron-donating group was present. Interestingly, the distribution of the product isomers in all cases of Table 2 was largely independent of the original alkyl structures.

**Table 2:** Synthesis of higher carboxylic acids using various ether substrates.

Reaction conditions: 20 μmol IrI_4_, 2 mmol LiI, 0.6 mL AcOH, 2.45 mmol substrate, 5 MPa CO_2_ (68 mmol) and 2 MPa H_2_ (at room temperature), 170 °C, 16 h

^a^Yield denotes 100 × moles of products per mole of ether feedstock

^b^1.23 mmol substrate was used because there are two alkyl groups in each molecule

^c^1.5 mmol substrate was used because of the large molar volume

### Reaction mechanism

**Fig. 3:**
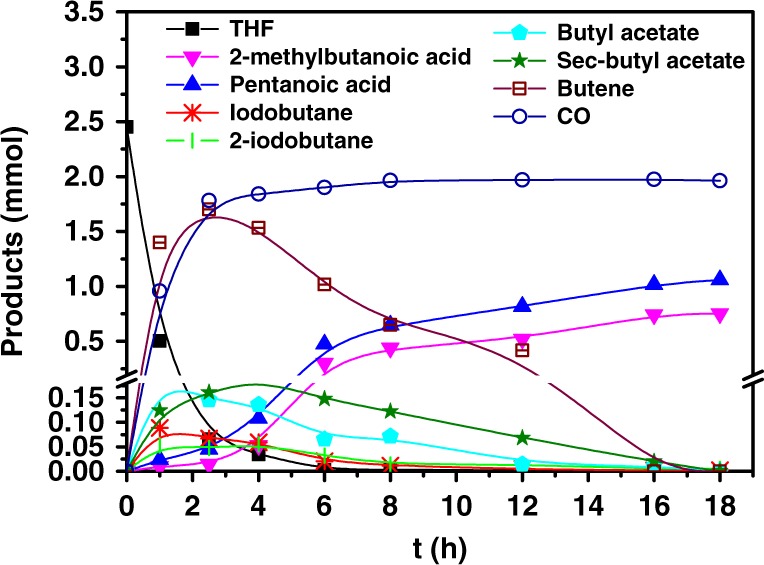
Time course of the reaction. Reaction conditions: 20 μmol IrI_4_, 2 mmol LiI, 0.6 mL AcOH, 2.45 mmol THF, 5 MPa CO_2_ (68 mmol) and 2 MPa H_2_ (at room temperature), 170 °C.

Figure 3 depicts the time course of the reaction. Interestingly, THF was quickly and high selectively converted to butene, including 1-butene, cis-2-butene and trans-2-butene, at the beginning of the reaction (Supplementary Fig. 34). The butene was further transformed into butyl/sec-butyl acetates and/or iodobutane/2-iodobutane. After the emergence of the acetates and alkyl iodides, the C_5_ carboxylic acids, i.e., pentanoic acid and 2-methylbutanoic acid, were observed and their amount increased rapidly. The amount of butene dropped remarkably with the reaction going on, while the amount of acetates and alkyl iodides kept at a much lower level all the time. These phenomena suggested that THF was converted to C_5_ products via butene, butyl/sec-butyl acetates and/or iodides. Besides, CO appeared at the start of the reaction, and reached a stable level quickly. This indicated that CO was also a reactive intermediate. After 16 h, the amount of C_5_ carboxylic acids was unchanged because the intermediates derived from THF were nearly completely consumed.

Transformation of higher ethers to olefins is still a challenge at present stage, which usually requires high temperature (ca. 450–650 °C) or with low yield (below 30%)^23,24^. Very interestingly, butene could be generated very efficiently at much lower temperature in our catalytic system. The high-efficiency generation of butene could be ascribed to the synergy of IrI_4_ and LiI (Supplementary Fig. 35). The control experiment using toluene as solvent suggested that the solvent acidity was not necessary in converting ether to olefins. Enlightened by the fact that butene engendered at the beginning of the reaction, we used cyclohexene as the substrate at similar condition, which is easier to handle. The result showed that cyclohexanecarboxylic acid was produced in high yield (60%). This supported that butene was a reactive intermediate of the reaction using THF as substrate. Figure 3 also suggests that the alkyl iodides and/or acetates were derived from butene. For longer chain olefins, the isomerization of the double bond usually occurred in the reaction, generating isomerized products^25^. This may account for the formation of butyl and sec-butyl intermediates from butene. Similar intermediates were also detected when other ether substrates were tested. The control experiments indicated that alkyl iodides and acetates could readily transform into each other at the reaction condition. To clarify whether the iodides or the acetates actually participated in the C–C bond formation, we conducted systematic control tests. The results confirmed that the iodides were indispensable in forming the C_5_ carboxylic acids (Supplementary Table 5). The alkyl acetates may act as a pool to store and protect the alkyl part of the ether during the reaction. Because the alkyl iodides were much easier to be reduced to alkanes in the presence of H_2_ (Supplementary Table 6). This indicates that the mechanism of olefin transformation in this work is different from that accelerated by Rh catalyst^18^.

As shown in Fig. 3, CO was in situ generated in the reaction. The control test without THF affirmed that CO was produced from CO_2_ via RWGS reaction (Supplementary Fig. 36). Further experiment showed that THF and CO (1 MPa) as reactants could efficiently generate target C_5_ carboxylic acids, and the product distribution was close to that using THF, CO_2_, and H_2_ (Supplementary Table 7, entry 1). The control test indicated that THF was also firstly converted to butene and then participated in further transformation. In contrast, the yield of the C_5_ carboxylic acids did not increase obviously when H_2_ was charged together with CO (entries 1, 3). Hence the major role of H_2_ was to produce CO in RWGS step. Although CO was a key reactive intermediate, too much of it would suppress the reaction due to the catalyst poisoning (entries 2–4). The results of ^13^CO_2_ and H_2_^18^O labeling tests demonstrated that the C = O and OH in the COOH group of the target carboxylic acids were from CO_2_ and H_2_O respectively (Supplementary Figs. 37–39). The GC-MS results of D_2_ and D_2_O labeling tests suggest that obvious H-D exchange existed during the reaction (Supplementary Figs. 40 and 41). The detailed study of such exchange reactions has been reported elsewhere^26^.

**Fig. 4:**
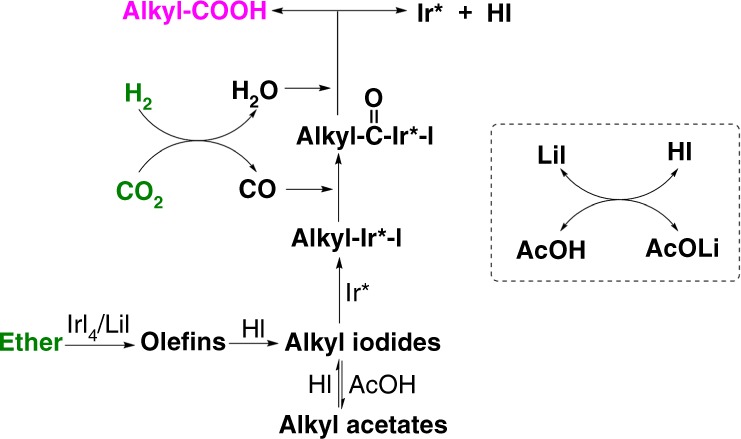
The proposed reaction mechanism. Ir* represents the active center of the Ir catalyst. The ether is converted to olefins, which are further transformed into alkyl iodides. The higher carboxylic acids are produced by carbonylation of alkyl iodides with CO generated in situ via RWGS reaction.

Based on the above results and discussion, we rendered the possible reaction mechanism (Fig. 4). Firstly, ether substrate is quickly and selectively converted to olefin, which is key to success of this route. The olefin is further converted into alkyl iodides and/or acetates, which can readily transform into each other. Alkyl iodides participate in the C–C bond formation step, while the alkyl acetates act as a temporary pool of the alkyl intermediates. Then oxidative addition of the alkyl iodides to Ir catalyst takes place, which is followed by insertion of CO generated via RWGS reaction. Next step is the reductive elimination of Alkyl-C = O (acyl) in the presence of water formed in situ, where the C_1_-elongated carboxylic acids are produced. Alkyl iodides generally undergo oxidative addition much more readily than alkyl chlorides or bromides, which accounts for the better performance of LiI than LiCl or LiBr. The oxidative addition of alkyl iodide, CO insertion, and reductive elimination of acyl are basic steps in organic reactions catalyzed by transition metal catalysts^27–30^. Although the Ir catalyzed methanol carbonylation that manufactures acetic acid from methanol and CO has been well studied^27,28^, our paper is different from that technology not only in reactants and products but also in catalyst system and reaction pathway. When the ethers with shorter alkyl chains were used as substrate, C_1_-elongated olefins were observed at the beginning of the reaction, which accounts for the formation of C_2_-elongated carboxylic acids (Supplementary Fig. 42). The control experiments suggest that CO_2_ also takes part in producing the C_1_-elongated olefin intermediates. To better understand the reaction mechanism, we studied the structure of the catalyst after the reaction. The FTIR characterization of the liquid sample demonstrates that cis-[Ir(CO)_2_I_4_]^−^ existed in the reaction solution (Supplementary Fig. 43). The HR-ESI(-)-MS spectra reveal that different [Ir(CO)_x_I_y_]^−^ (x = 1–2, y = 2–4) species were observed, which may be caused by fragmentation of cis-[Ir(CO)_2_I_4_]^−^ at the testing condition (Supplementary Fig. 44).

The catalytic behavior and the catalyst structure characterization after the reaction suggested that the catalyst was stable in the reaction, indicating that the catalyst has potential of reuse. The major obstacle for recycling the homogeneous catalyst is separation of catalyst from the reaction product, and the effective method should be developed for practical application. To study whether our catalytic system can be translated into a heterogeneous mode, we conducted experiments using different heterogeneous Ir catalysts (Supplementary Table 8). The results indicated that activated carbon supported Ir catalyst (Ir/AC) could accelerate the reaction, but the efficiency was much lower than that of the homogeneous ones.

In summary, we have developed a route to synthesize higher carboxylic acids from ethers, CO_2_ and H_2_. The reaction can be effectively accelerated by a homogeneous Ir catalyst. The higher carboxylic acids can be produced at above 130 °C and high yields are obtained at optimized conditions. The catalyst has very good substrate adaptability and various kinds of ethers, such as cyclic ethers, n-alkyl ethers, iso-alkyl ethers and aryl alkyl ethers, can be efficiently converted to corresponding higher carboxylic acids. The target products are produced via tandem reactions. The ether substrate is quickly and selectively transformed into olefin, which is key to the success of this strategy. The olefin is further converted into alkyl iodides and/or acetates. The alkyl iodides participate in the C–C bond formation in the presence of CO generated by RWGS reaction, while the alkyl acetates act as a temporary pool of alkyl part of the ether. The C_1_-elongated carboxylic acids are produced by reductive elimination of acyl in the presence of water formed in situ. This route has great potential of application because cheap and easily available feedstocks are utilized with high efficiency. We believe that this work will trigger more research for CO_2_ transformation into value-added chemicals.

## Methods

### General information

All reagents were commercially supplied and used as received. All the reactions were carried out in a 16 mL Teflon-lined stainless steel batch reactor equipped with a magnetic stirrer. The inner diameter of the reactor was 18 mm. The amount of carboxylic acids in the reaction solution were determined by a liquid chromatograph (LC-10AT, SHIMADZU) equipped with a refractive index detector (RID) and a BP-800 H^+^ carbohydrate column (S/N 23757, Benson polymeric) using dioxane or dimethyl sulfoxide (DMSO) as the internal standard. The column temperature was maintained at 323.15 K. The column was eluted with 5 mmol/L H_2_SO_4_ solution at a flow rate of 0.5 ml/min or 1.0 ml/min according to the carboxylic acids analyzed. The reactive intermediates (butyl/sec-butyl acetates and iodobutane/2-iodobutane) in the solution were determined using a gas chromatograph (GC, Agilent 7890B) equipped with a flame ionization detector (FID) and an HP-INNOWAX capillary column (length = 30 m, diameter = 0.25 mm, film = 0.25 μm) using dodecane as the internal standard. The reaction products were identified by GC-MS (SHIMADZU GC-MS-QP2010) using Rtx-WAX column (length = 30 m, diameter = 0.32 mm, film = 0.25 μm) as well as comparing the retention time with the respective standard in the LC or GC traces. The gaseous samples after reaction were detected by a GC (Agilent 7890B) equipped with a TCD detector and a packed column (carbon molecular sieve TDX-01, 3 mm in diameter and 1 m in length) using argon as the carrier gas. The olefins in gaseous sample was determined using a gas chromatograph (GC, Agilent 7890B) equipped with a flame ionization detector (FID) and a GS-Gaspro capillary column (length = 30 m, diameter = 0.32 mm). The amount of butene during the reaction was calculated according to conservation of matter. Liquid ^13^C NMR spectra were recorded on Bruck Avance III 400 HD NMR spectrometer. NMR chemical shifts δ are given in parts per million (ppm) relative to tetramethylsilane. Infrared Radiation (IR) spectroscopy were recorded on Bruker Tensor-27 FT-IR Spectrometer. High-resolution electrospray ionization mass spectrometry (HR-ESI-MS) were recorded on Thermo-Exactive. The needle voltage was 3.5 kV.

### Catalytic reaction

Before the reaction, the IrI_4_ catalyst, the LiI promoter, the THF substrate and the AcOH solvent were added into the reactor. The above components could be substituted by others if needed. The reactor was sealed and 0.5 MPa of CO_2_ was utilized to replace the air in the reactor for two times. Then CO_2_ and H_2_ of specified pressure at ambient temperature were charged into the reactor, respectively. The reactor was placed in a furnace and was heated to constant temperature, where the stirrer rotated at 800 rpm. When the reaction was finished the reactor was put into an ice-water bath. After cooling, the residual gas in the reactor was discharged, during which the gaseous sample was collected in a gas bag. For LC analysis, the liquid mixture was diluted with 10 mL 1/1 AcOH/H_2_O, followed by addition of 10 µL dioxane or DMSO as the internal standard. For GC analysis, the liquid mixture was diluted with 4 mL acetone, followed by addition of 10 µL dodecane as the internal standard. Before LC or GC, an aliquot of the sample was filtered through syringe filters with hydrophilic PTFE membrane, and the filtrate was immediately injected into the chromatograph.

## Supplementary information

Supplementary Information

## Data Availability

All data supporting the findings of this study are available within the article, as well as the Supplementary Information file, or available from the corresponding authors on reasonable request.

## References

[1] He MY, Sun YH, Han BX (2013). Green carbon science: scientific basis for integrating carbon resource processing, utilization, and recycling. Angew. Chem. Int. Ed..

[2] Artz J (2018). Sustainable conversion of carbon dioxide: an integrated review of catalysis and life cycle assessment. Chem. Rev..

[3] Liu Q, Wu L, Jackstell R, Beller M (2015). Using carbon dioxide as a building block in organic synthesis. Nat. Commun..

[4] Liu Y, Zhou H, Guo JZ, Ren WM, Lu XB (2017). Completely recyclable mnomers and polycarbonate: approach to sustainable polymers. Angew. Chem. Int. Ed..

[5] Qian QL (2015). Highly selective hydrogenation of CO_2_ into C_2+_ alcohols by homogeneous catalysis. Chem. Sci..

[6] Klankermayer J, Wesselbaum S, Beydoun K, Leitner W (2016). Selective catalytic synthesis using the combination of carbon dioxide and hydrogen: catalytic chess at the interface of energy and chemistry. Angew. Chem. Int. Ed..

[7] Jessop PG, Joó F, Tai CC (2004). Recent advances in the homogeneous hydrogenation of carbon dioxide. Coord. Chem. Rev..

[8] Jessop PG, Ikariya T, Noyori R (1994). Homogeneous catalytic hydrogenation of supercritical carbon dioxide. Nature.

[9] Rohmann K (2016). Hydrogenation of CO_2_ to formic acid with a highly active ruthenium acriphos complex in DMSO and DMSO/water. Angew. Chem. Int. Ed..

[10] Moret S, Dyson PJ, Laurenczy G (2014). Direct synthesis of formic acid from carbon dioxide by hydrogenation in acidic media. Nat. Commun..

[11] Wu XF, Zheng F (2017). Synthesis of carboxylic acids and esters from CO_2_. Top. Curr. Chem. (Z.).

[12] Yu D, Teong SP, Zhang Y (2015). Transition metal complex catalyzed carboxylation reactions with CO_2_. Coord. Chem. Rev..

[13] Tortajada A, Julia-Hernandez F, Borjesson M, Moragas T, Martin R (2018). Transition metal catalyzed carboxylation reactions with carbon dioxide. Angew. Chem. Int. Ed..

[14] Juliá-Hernández F, Moragas T, Cornella J, Martin R (2017). Remote carboxylation of halogenated aliphatic hydrocarbons with carbon dioxide. Nature.

[15] Fujihara T, Nogi K, Xu T, Terao J, Tsuji Y (2012). Nickel-catalyzed carboxylation of aryl and vinyl chlorides employing carbon dioxide. J. Am. Chem. Soc..

[16] Sasano K, Takaya J, Iwasawa N (2013). Palladium(II)-catalyzed direct carboxylation of alkenyl C-H bonds with CO_2_. J. Am. Chem. Soc..

[17] Shi M, Nicholas KM (1997). Palladium-catalyzed carboxylation of allyl stannanes. J. Am. Chem. Soc..

[18] Ostapowicz TG, Schmitz M, Krystof M, Klankermayer J, Leitner W (2013). Carbon dioxide as a C1 building block for the formation of carboxylic acids by formal catalytic hydrocarboxylation. Angew. Chem. Int. Ed..

[19] Qian QL, Zhang JJ, Cui M, Han BX (2016). Synthesis of acetic acid via methanol hydrocarboxylation with CO_2_ and H_2_. Nat. Commun..

[20] Cui M, Qian QL, Zhang JJ, Chen CJ, Han BX (2017). Efficient synthesis of acetic acid via Rh catalyzed methanol hydrocarboxylation with CO_2_ and H_2_ under milder conditions. Green. Chem..

[21] Besson M, Gallezot P, Pinel C (2014). Conversion of biomass into chemicals over metal catalysts. Chem. Rev..

[22] Maitlis PM, Haynes A, James BR, Catellani M, Chiusoli GP (2004). Iodide effects in transition metal catalyzed reactions. Dalton. Trans..

[23] Yarbrough, C. M., Swarup, V., Maher, P. J., Hu, A. Y. & Bedell, M. W. Making olefin from dialkyl ether, comprises introducing ether into thermal or catalytic unit that processes hydrocarbon feedstock, and decomposing ether to form olefin and alcohol. US patent 7, 655,826 (2010).

[24] Keskivali J, Parviainen A, Lagerblom K, Repo T (2018). Transition metal triflate catalyzed conversion of alcohols, ethers and esters to olefins. RSC Adv..

[25] Wiese KD, Obst D (2006). Hydroformylation. Top. Organomet. Chem..

[26] Junk T, Catallo WJ (1997). Hydrogen isotope exchange reactions involving C-H (D,T) bonds. Chem. Soc. Rev..

[27] Maitlis PM, Haynes A, Sunley GJ, Howard MJ (1996). Methanol carbonylation revisited: thirty years on. J. Chem. Soc., Dalton Trans..

[28] Haynes A (2004). Promotion of iridium-catalyzed methanol carbonylation: mechanistic studies of the Cativa process. J. Am. Chem. Soc..

[29] Haynes A, Mann BE, Gulliver DJ, Morris GE, Maitlis PM (1991). Direct observation of MeRh(CO)_2_I_3_^−^, the key intermediate in rhodium-catalyzed methanol carbonylation. J. Am. Chem. Soc..

[30] Watanabe K, Kudo K, Sugtta N (1985). Kinetics and mechanistic study of the methanol homologation with cobalt-ruthenium mixed catalyst. Bull. Chem. Soc. Jpn..

