# Supplementary material for: Synthesis of higher carboxylic acids from ethers, CO2 and H2
Source: Nat Commun. 2019 Dec 4;10:5395. doi: 10.1038/s41467-019-13463-0 (PMC6892813; doi:10.1038/s41467-019-13463-0)
Supplement: Supplementary file 1 — Supplementary Information [file 41467_2019_13463_MOESM1_ESM.pdf]

Supplementary Information

Synthesis of higher carboxylic acids from ethers, CO_2 and H_2

Wang et al.

Supplementary Figures

Entry 1 (THF)

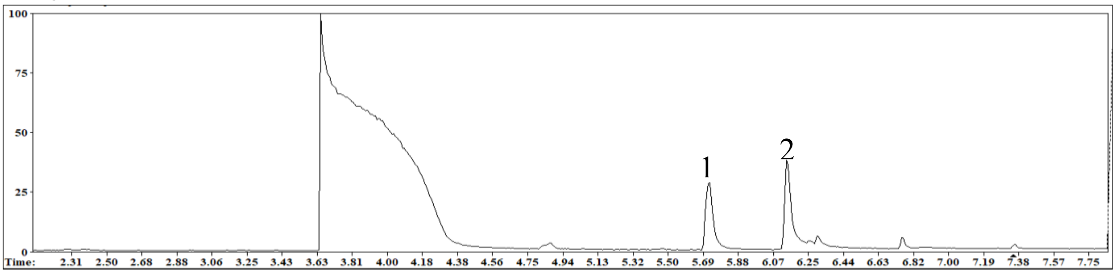

Peak 1:

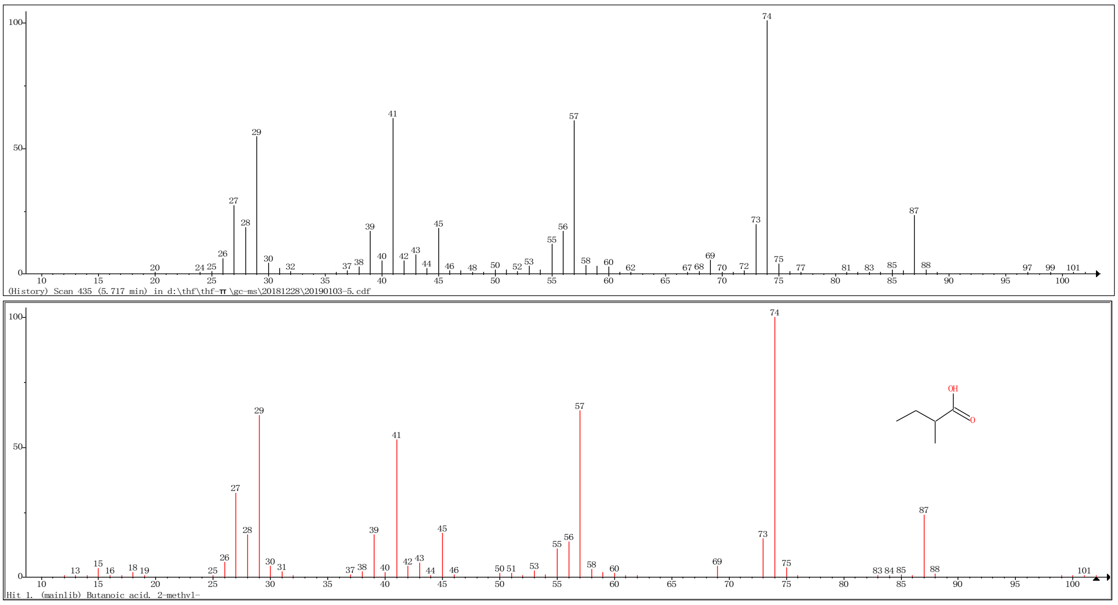

Peak 2:

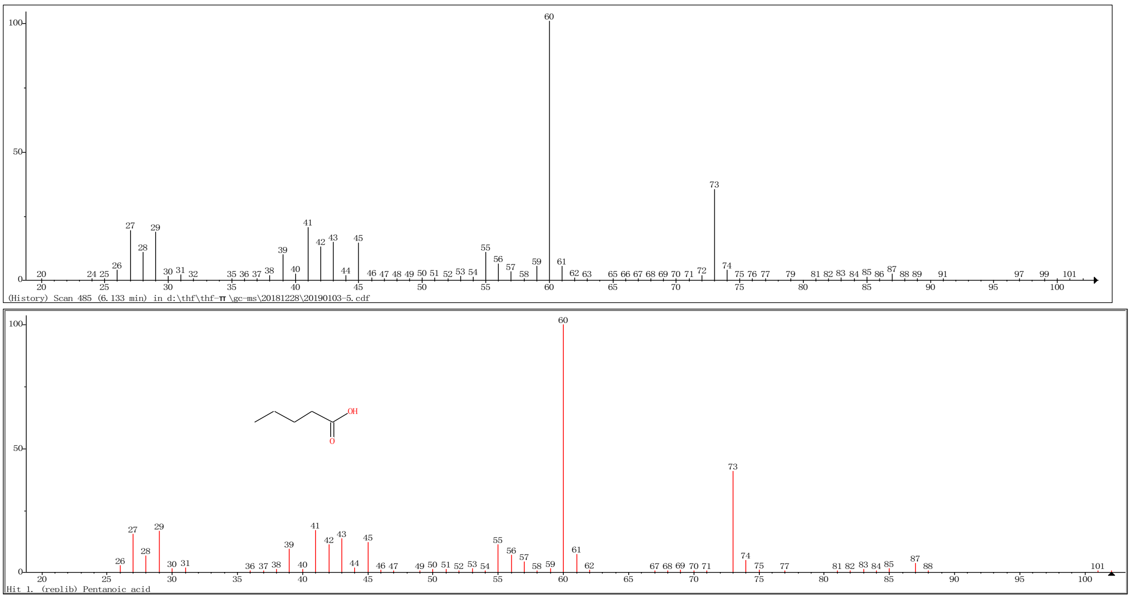

Supplementary Fig. 1 The GC-MS spectra of the reaction solution using THF as substrate.

Entry 2 (2-methyltetrahydrofuran)

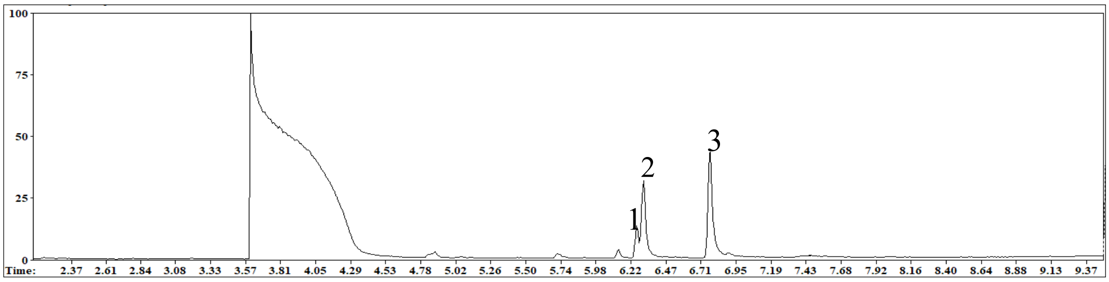

Peak 1：

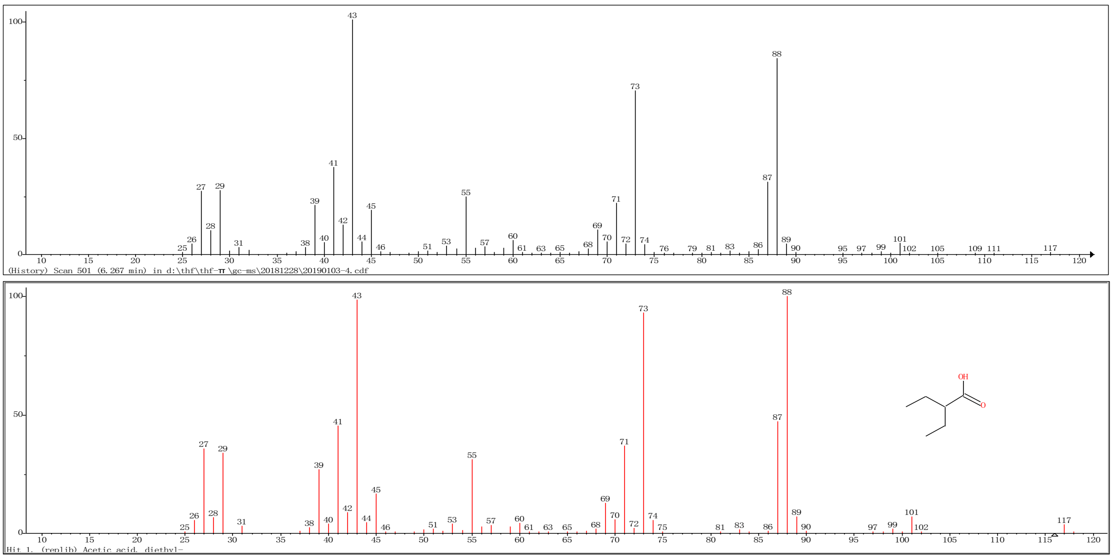

Peak 2：

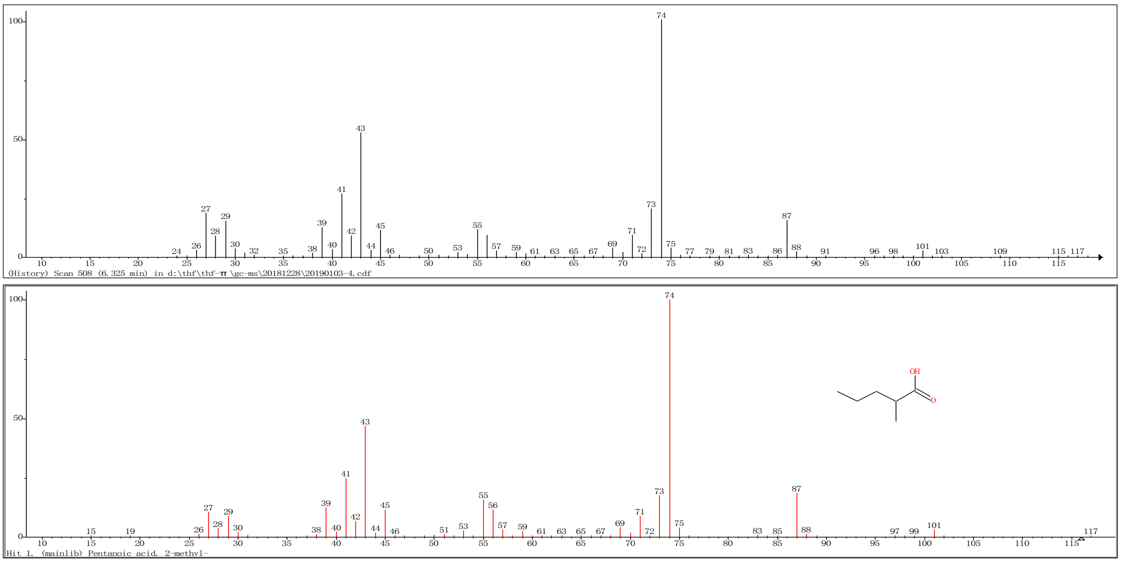

Peak 3:

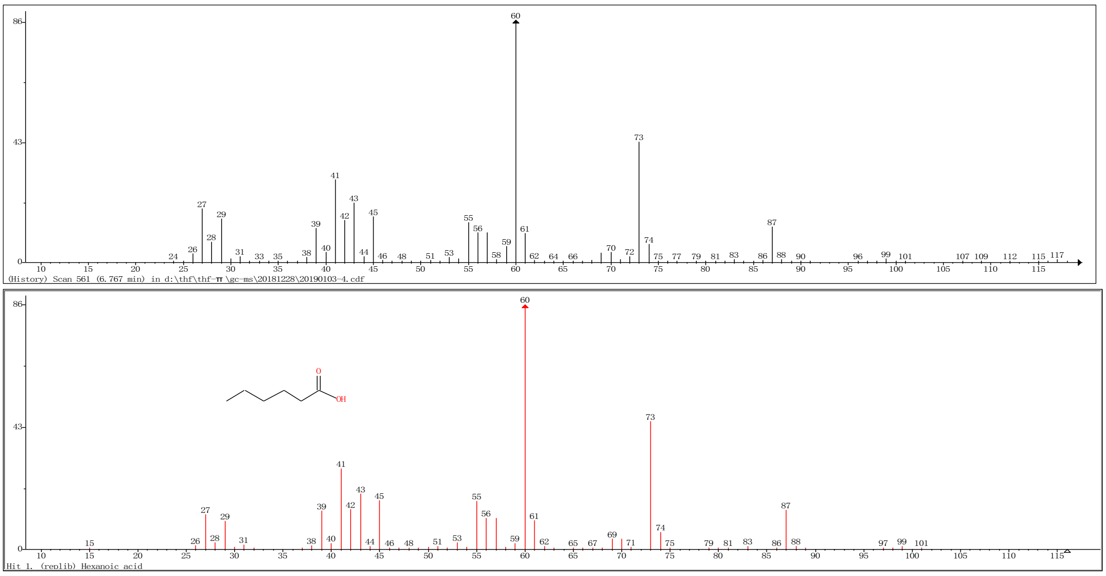

Supplementary Fig. 2 The GC-MS spectra of the reaction solution using 2-methyltetrahydro-furan as substrate.

Entry 3 (2,5-dimethyltetrahydrofuran)

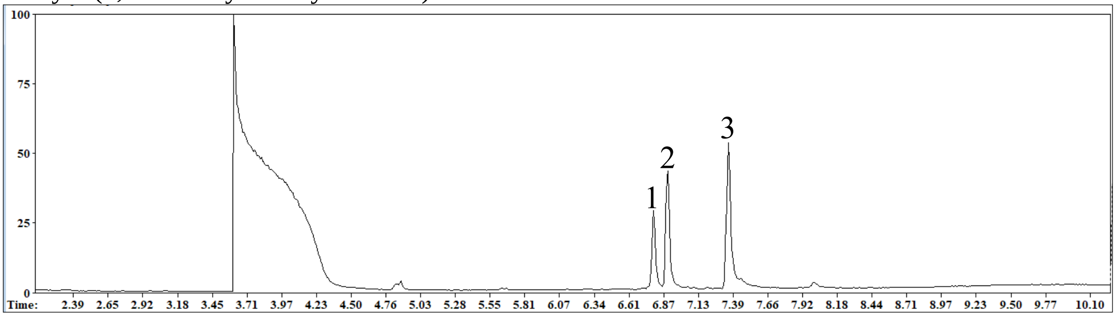

Peak 1:

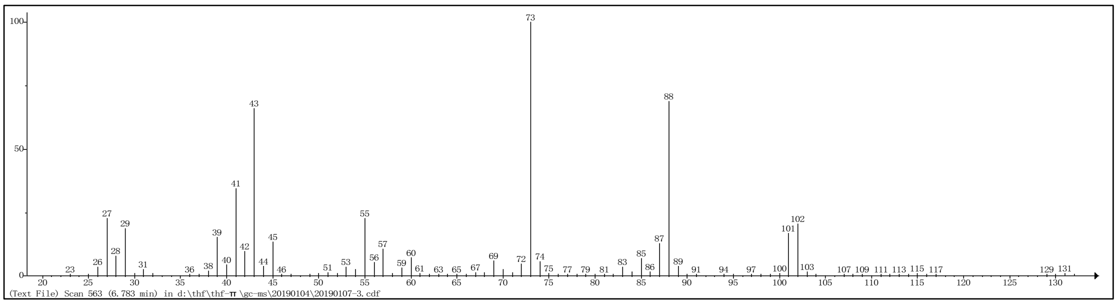

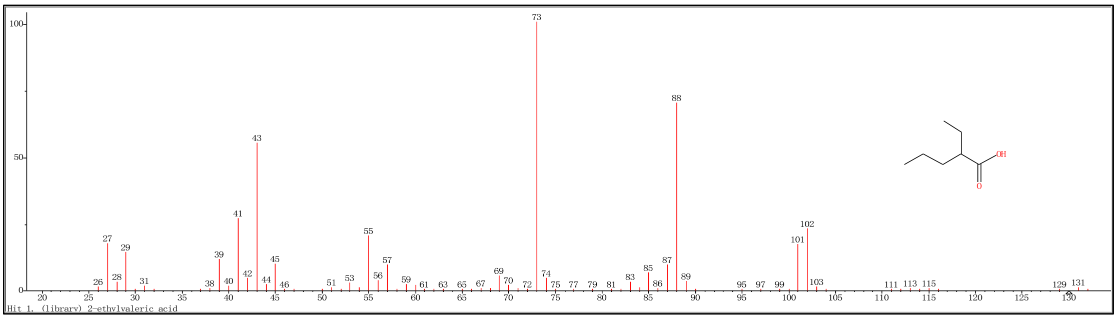

Peak 2:

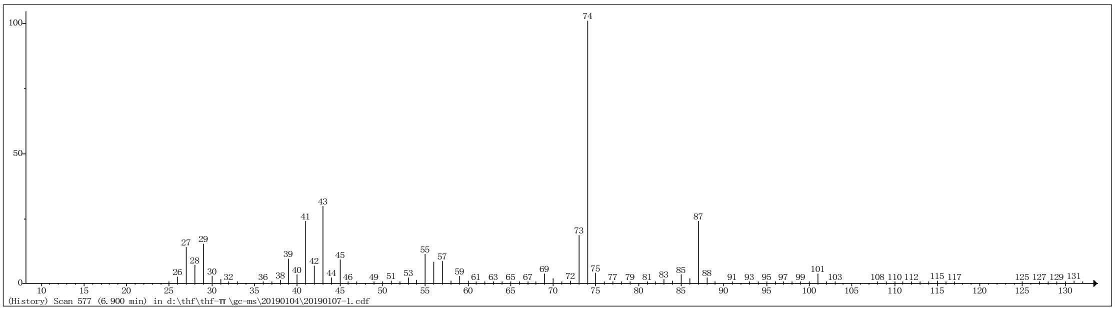

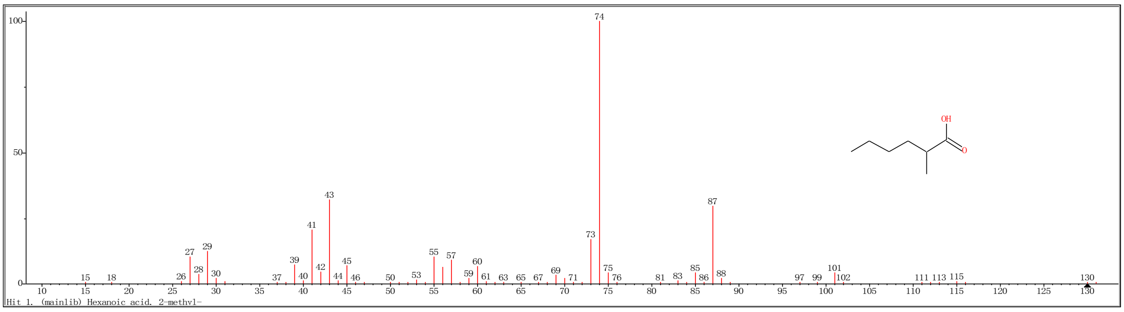

Peak 3:

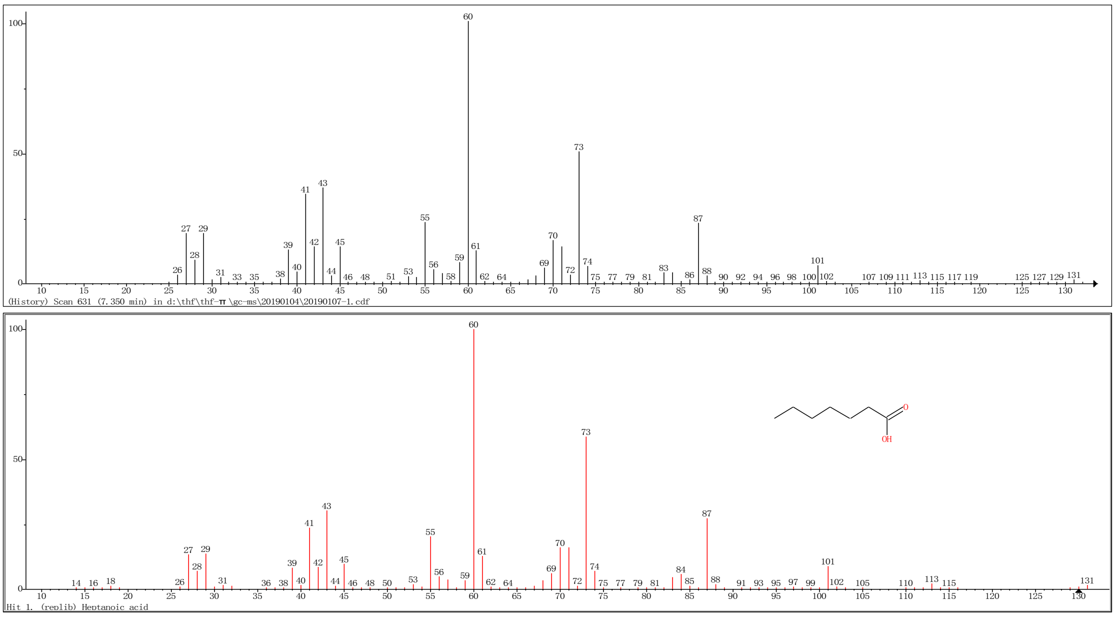

Supplementary Fig. 3 The GC-MS spectra of the reaction solution using 2,5-dimethyltetrahydro-furan as substrate.

Entry 5 (oxetane)

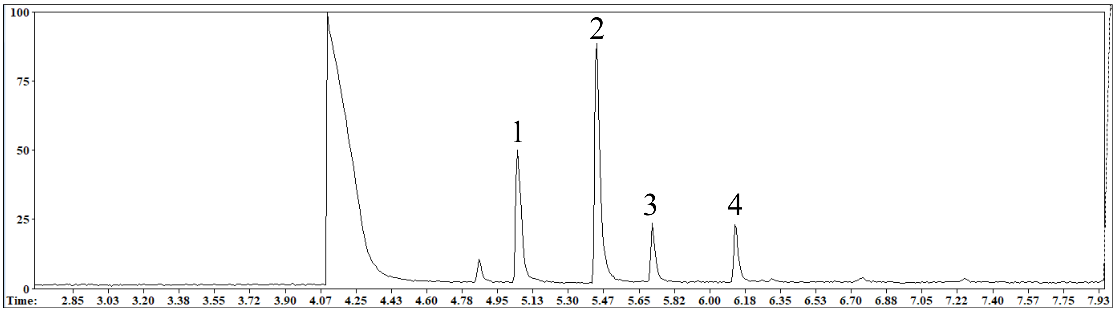

Peak 1:

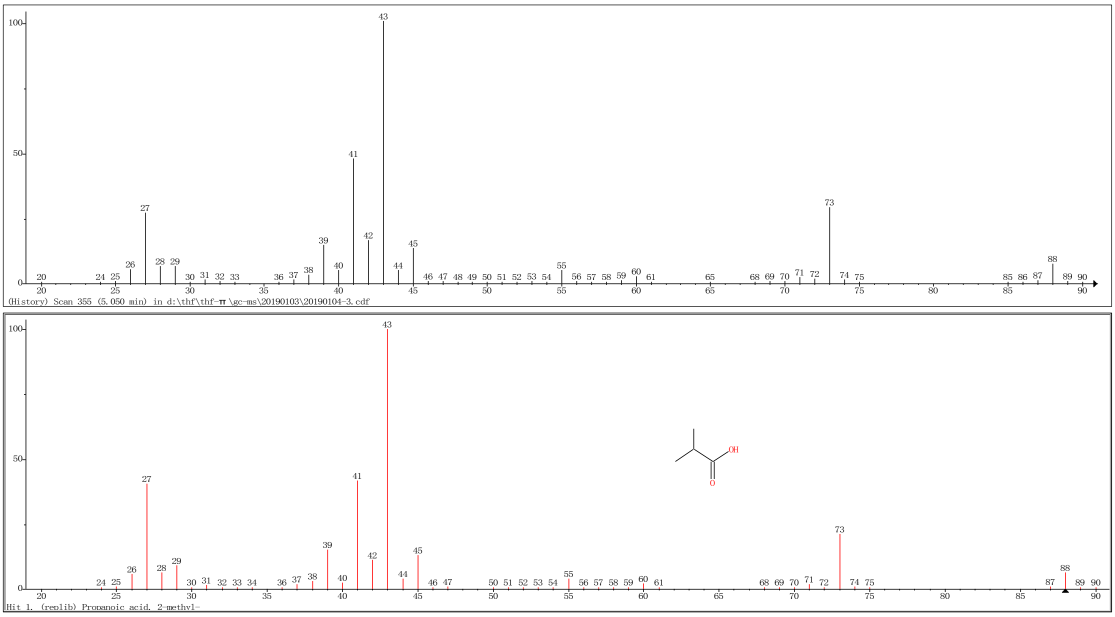

Peak 2:

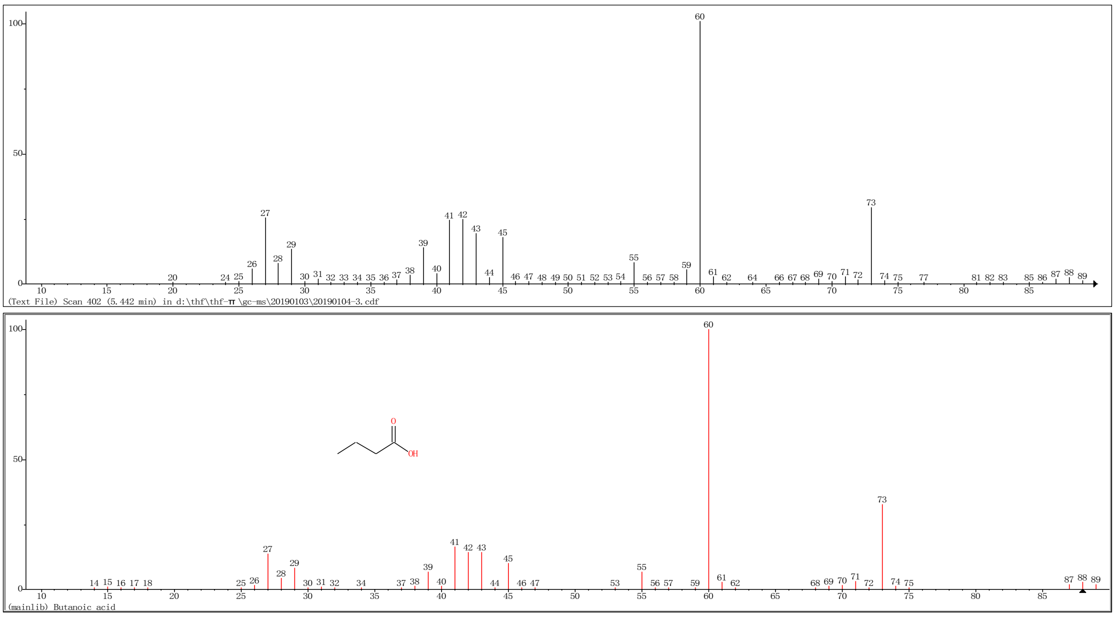

Peak 3：

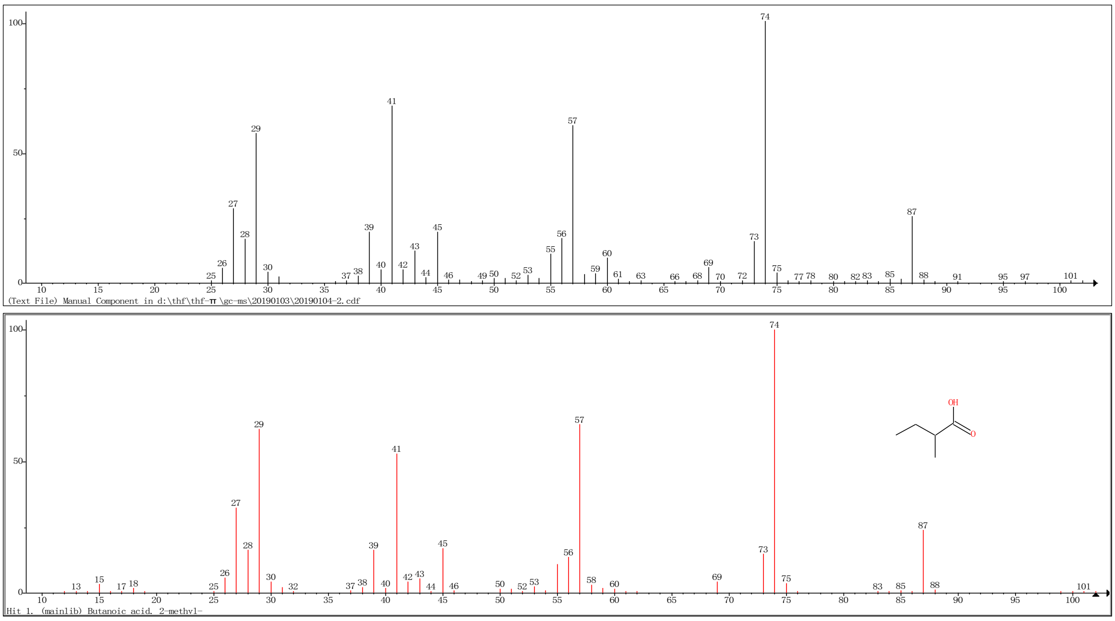

Peak 4：

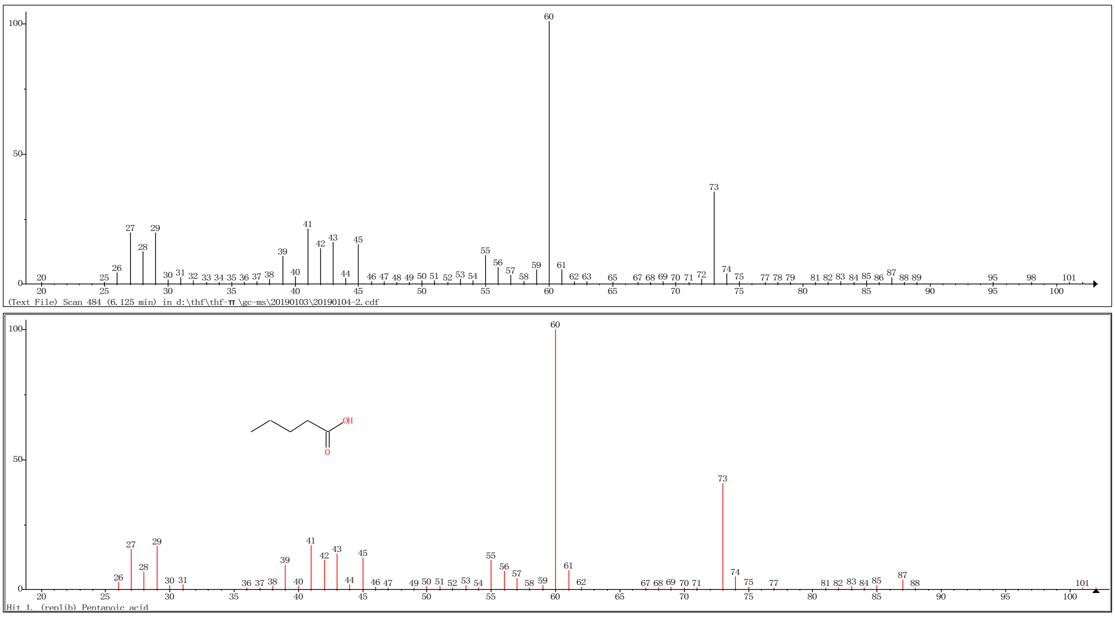

Supplementary Fig. 4 The GC-MS spectra of the reaction solution using oxetane as substrate.

Entry 9 (cyclopentene oxide)

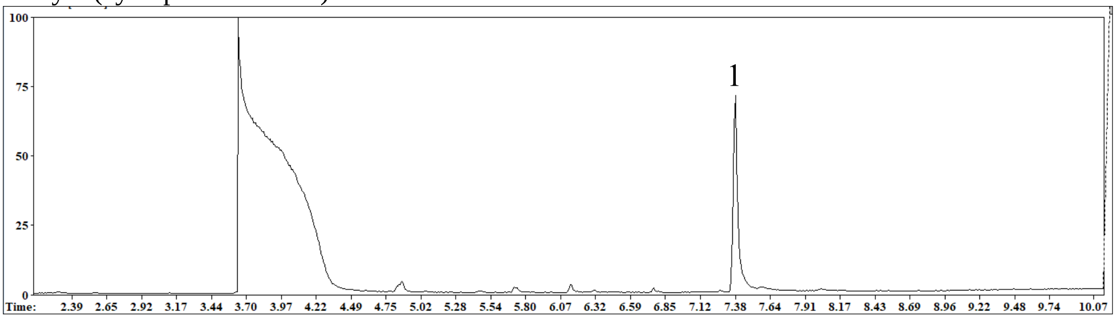

Peak 1:

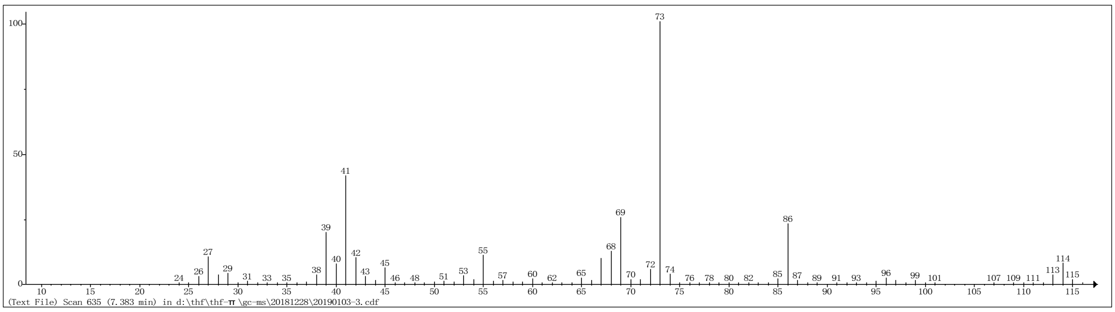

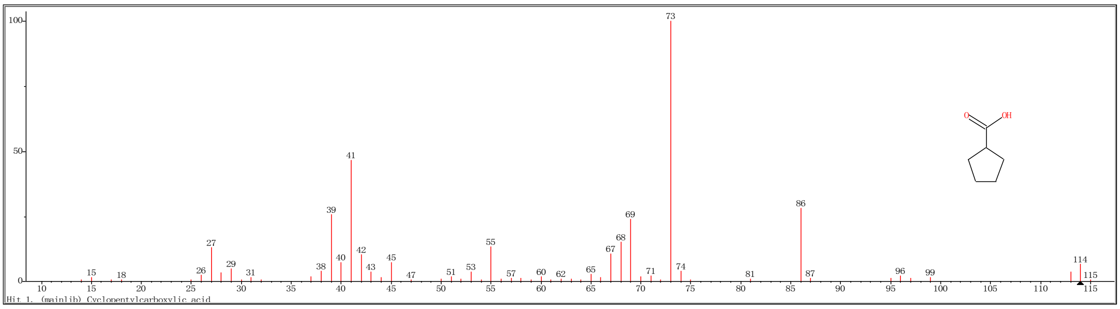

Supplementary Fig. 5 The GC-MS spectra of the reaction solution using cyclopentene oxide as substrate.

Entry 10 (cyclohexene oxide)

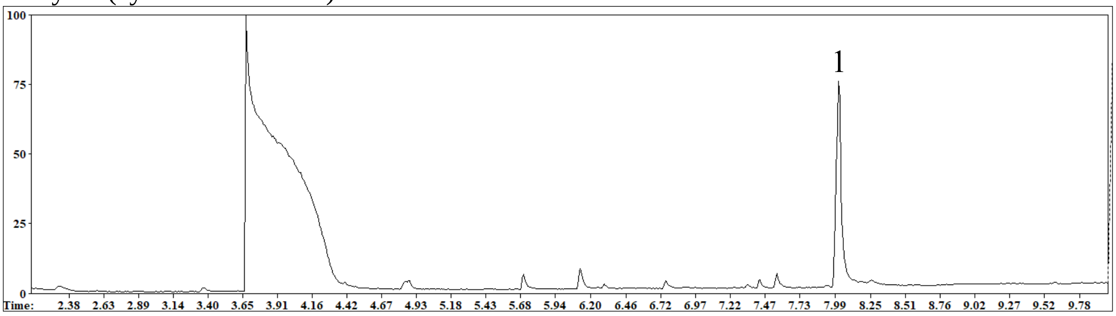

Peak 1:

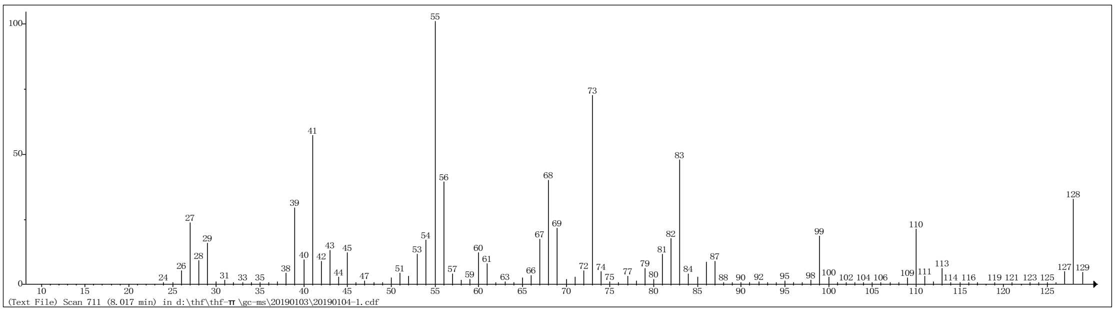

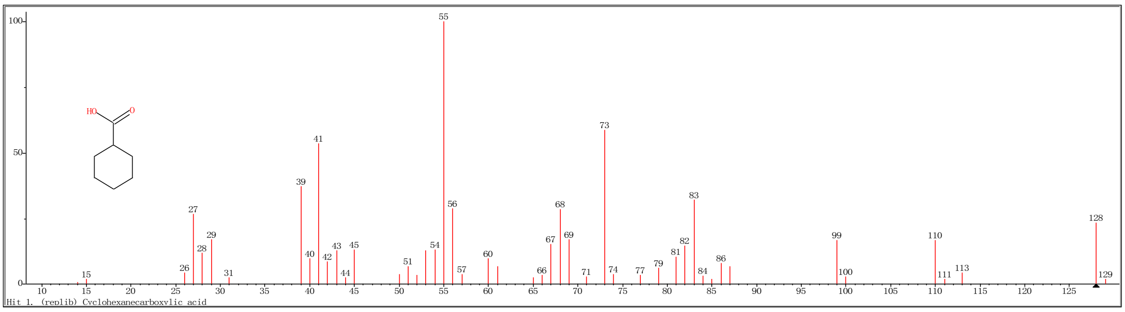

Supplementary Fig. 6 The GC-MS spectra of the reaction solution using cyclohexene oxide as substrate.

Entry 12 (diethyl ether)

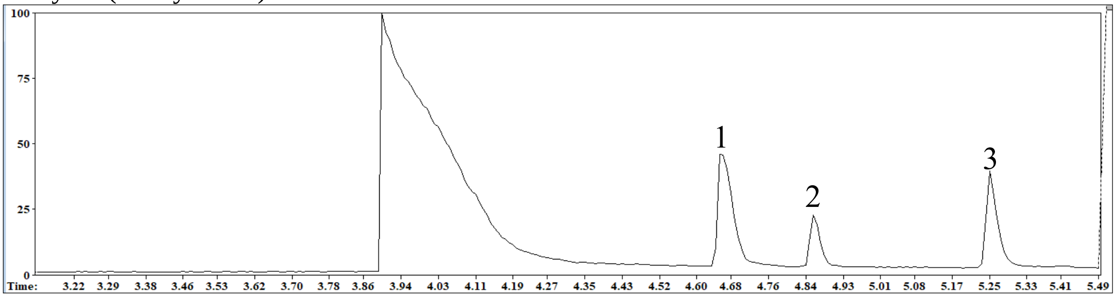

Peak 1：

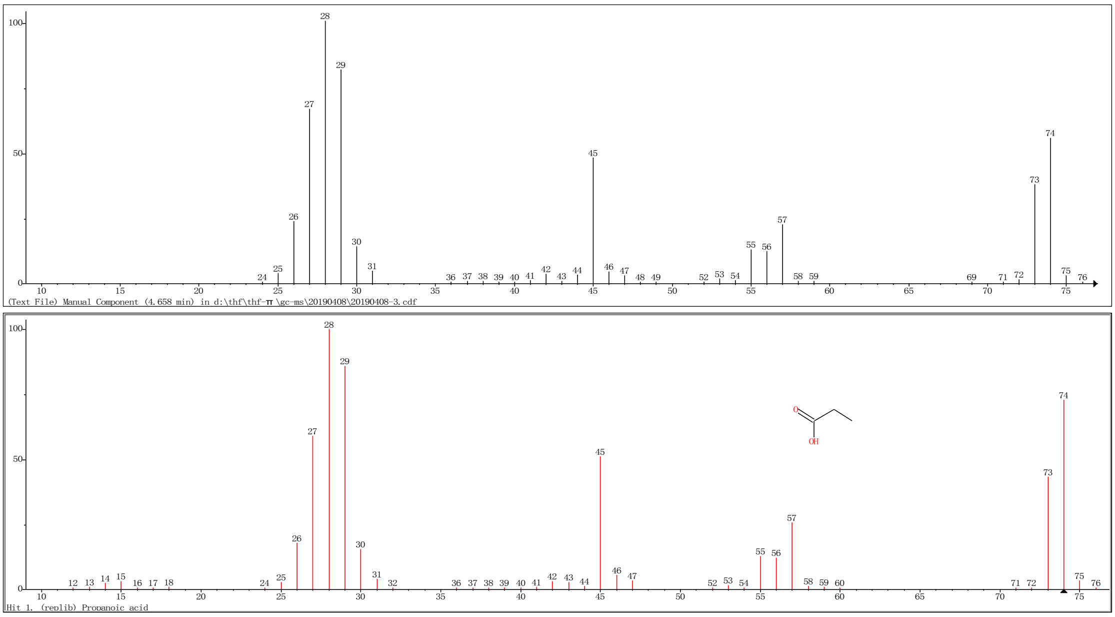

Peak 2：

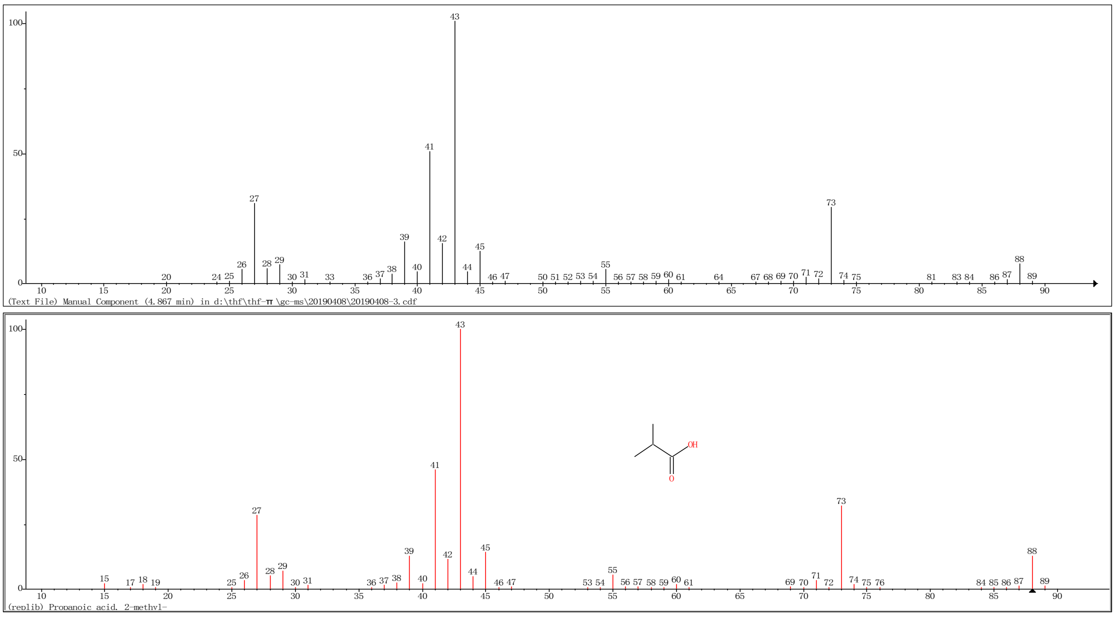

Peak 3：

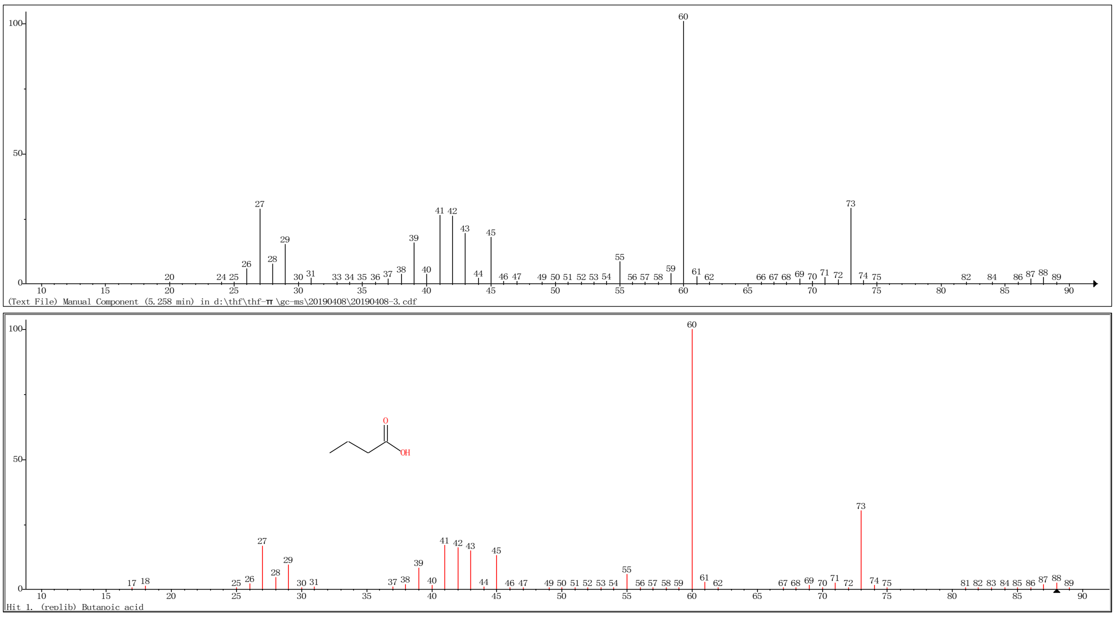

Supplementary Fig. 7 The GC-MS spectra of the reaction solution using diethyl ether as substrate.

Entry 1 (THF)

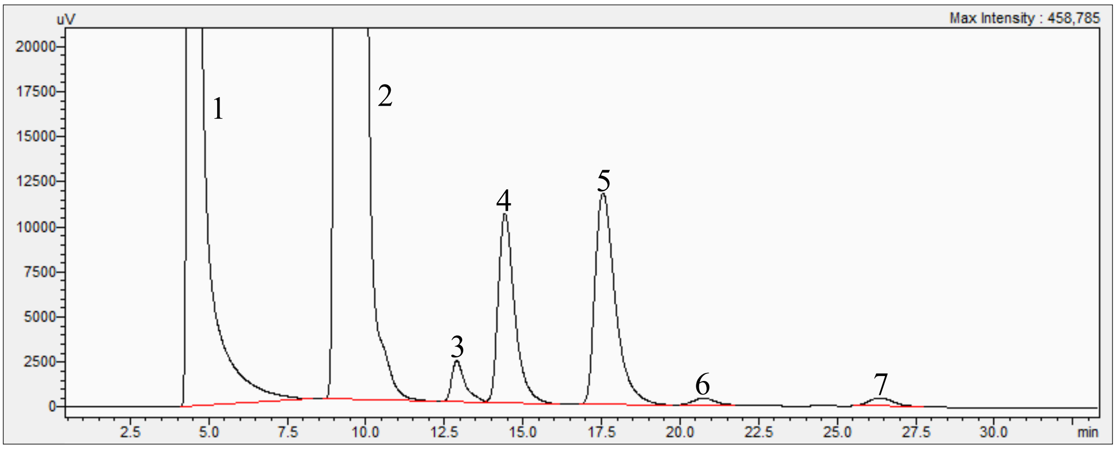

Supplementary Fig. 8 The LC graph of the reaction solution using THF as substrate.

Entry 2 (2-methyltetrahydrofuran)

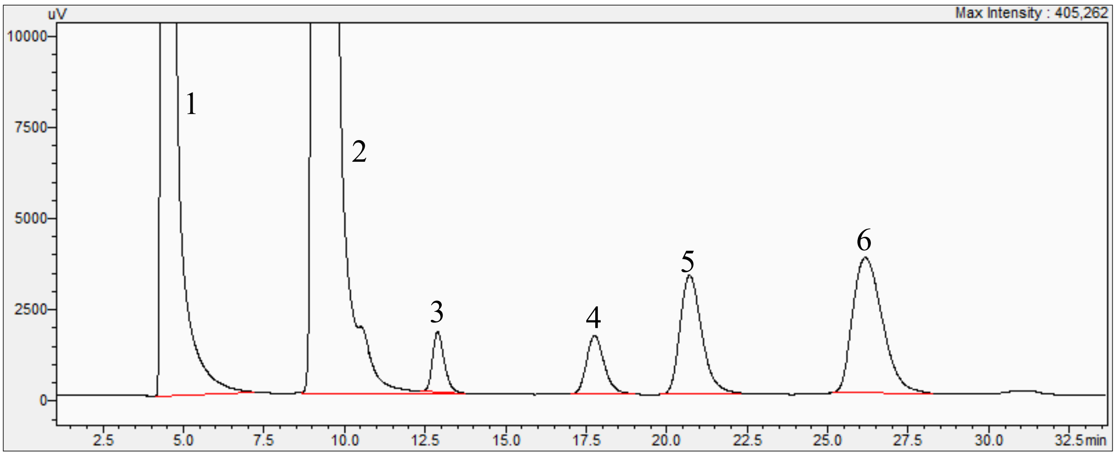

Supplementary Fig. 9 The LC graph of the reaction solution using 2-methyltetrahydrofuran as substrate.

Entry 3 (2,5-dimethyltetrahydrofuran)

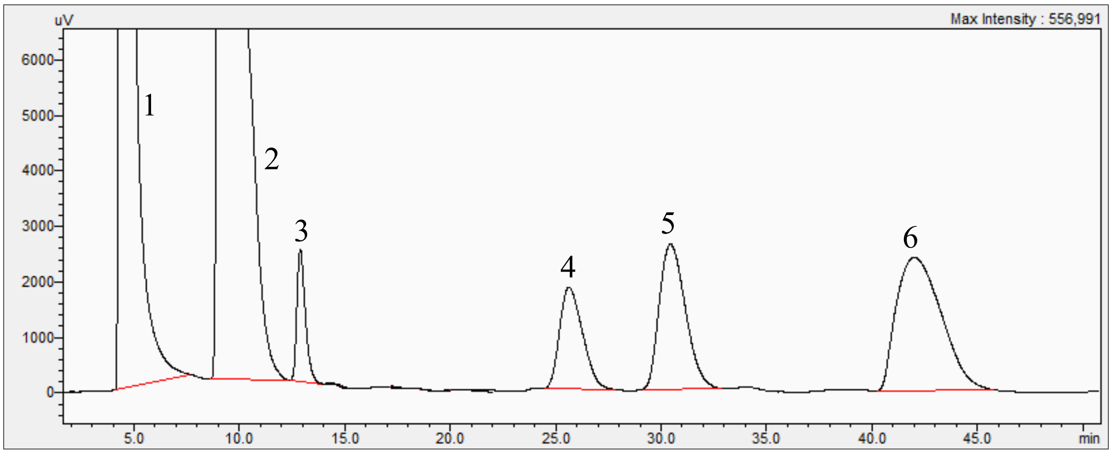

Supplementary Fig. 10 The LC graph of the reaction solution using 2,5-dimethyltetrahydrofuran as substrate.

Entry 4 (furan)

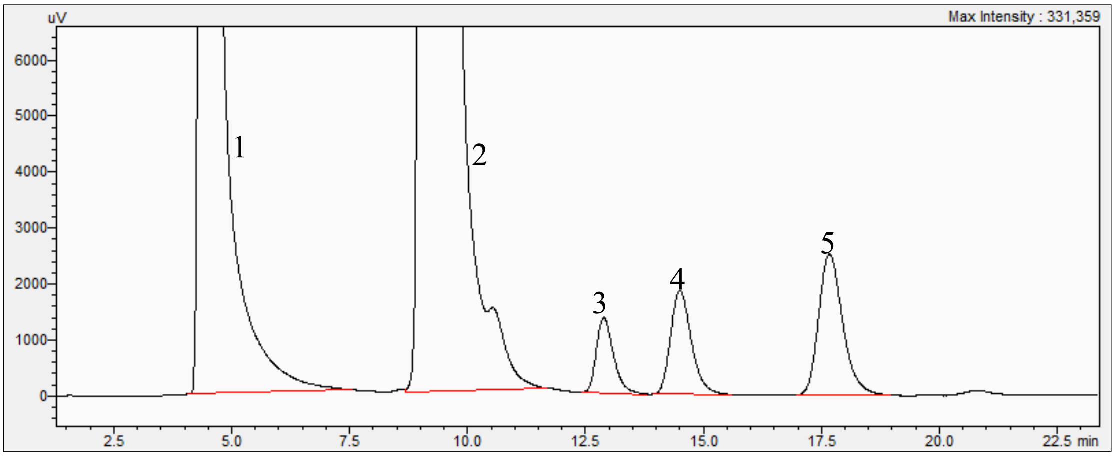

Supplementary Fig. 11 The LC graph of the reaction solution using furan as substrate.

Entry 5 (oxetane)

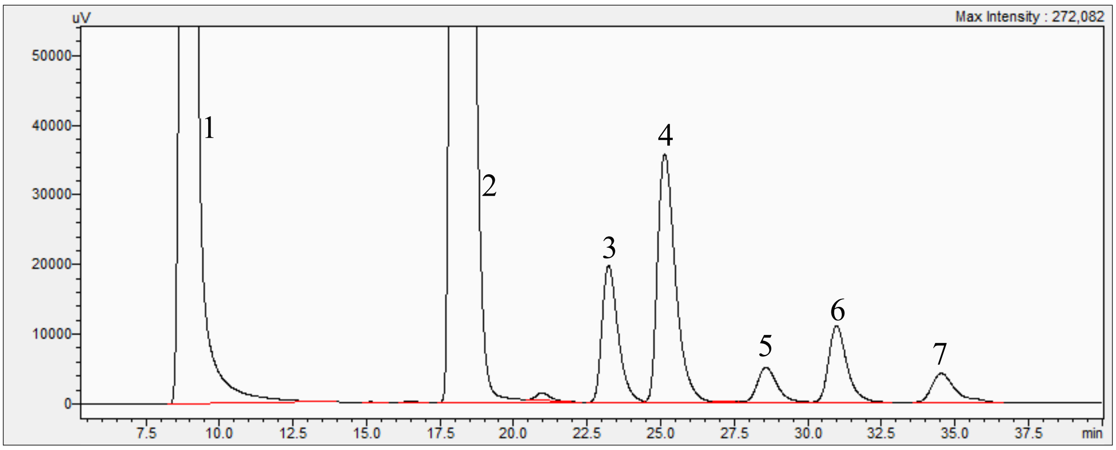

Supplementary Fig. 12 The LC graph of the reaction solution using oxetane as substrate.

Entry 6 (tetrahydropyran)

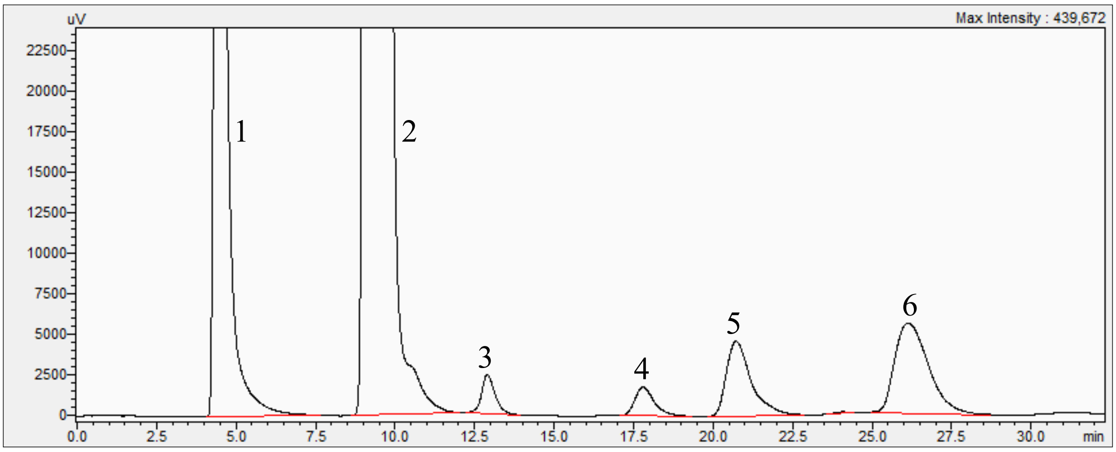

Supplementary Fig. 13 The LC graph of the reaction solution using tetrahydropyran as substrate.

Entry 7 (oxepane)

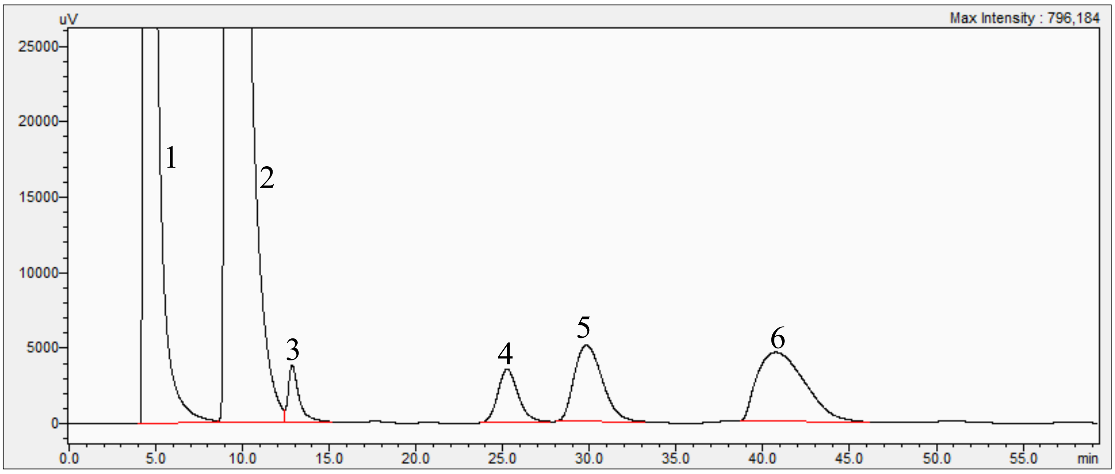

Supplementary Fig. 14 The LC graph of the reaction solution using oxepane as substrate.

Entry 8 (propylene oxide)

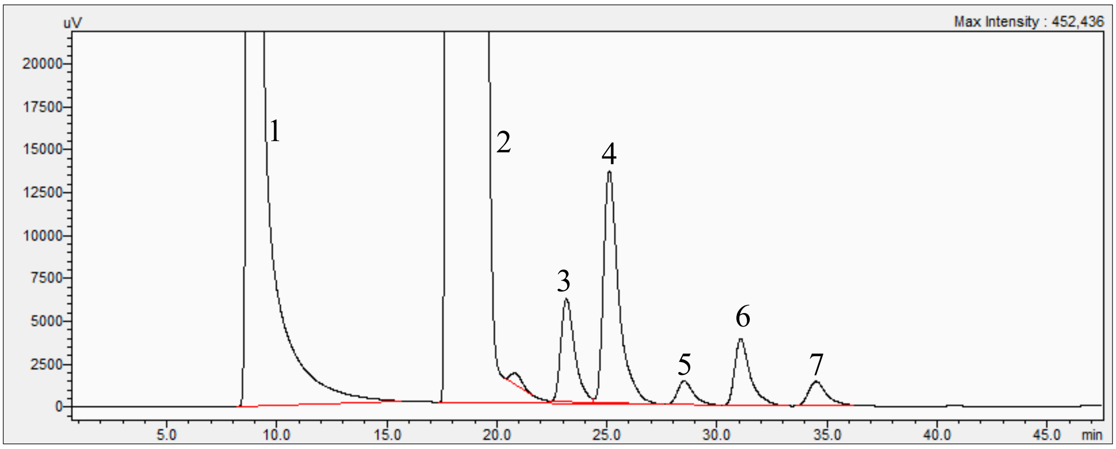

Supplementary Fig. 15 The LC graph of the reaction solution using propylene oxide as substrate.

Entry 9 (cyclopentene oxide)

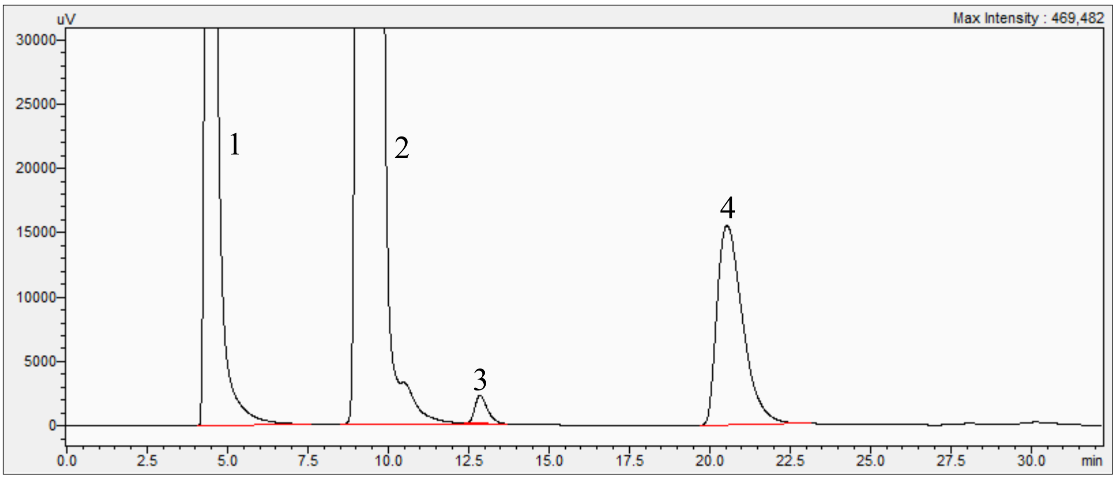

Supplementary Fig. 16 The LC graph of the reaction solution using cyclopentene oxide as substrate.

Entry 10 (cyclohexene oxide)

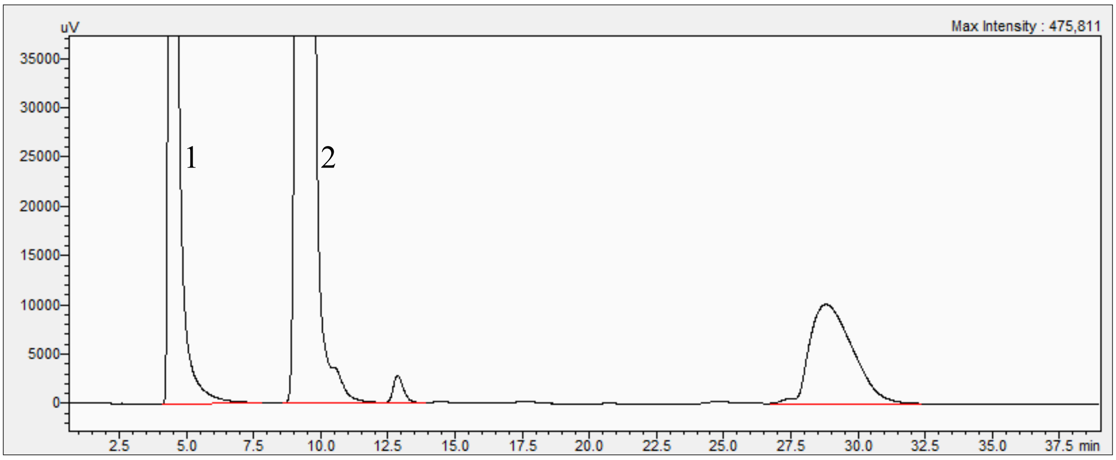

Supplementary Fig. 17 The LC graph of the reaction solution using cyclohexene oxide as substrate.

Entry 11 (dioxane)

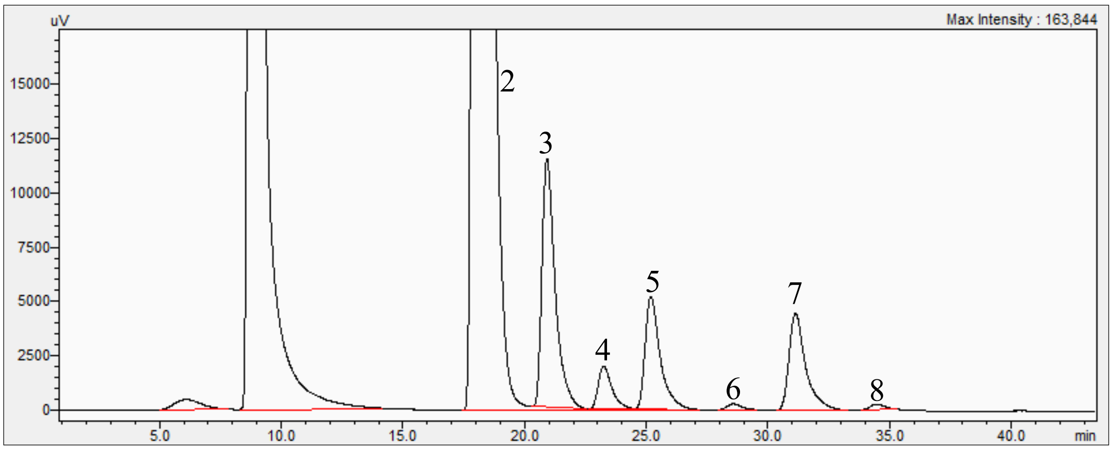

Supplementary Fig. 18 The LC graph of the reaction solution using dioxane as substrate.

Entry 12 (diethyl ether)

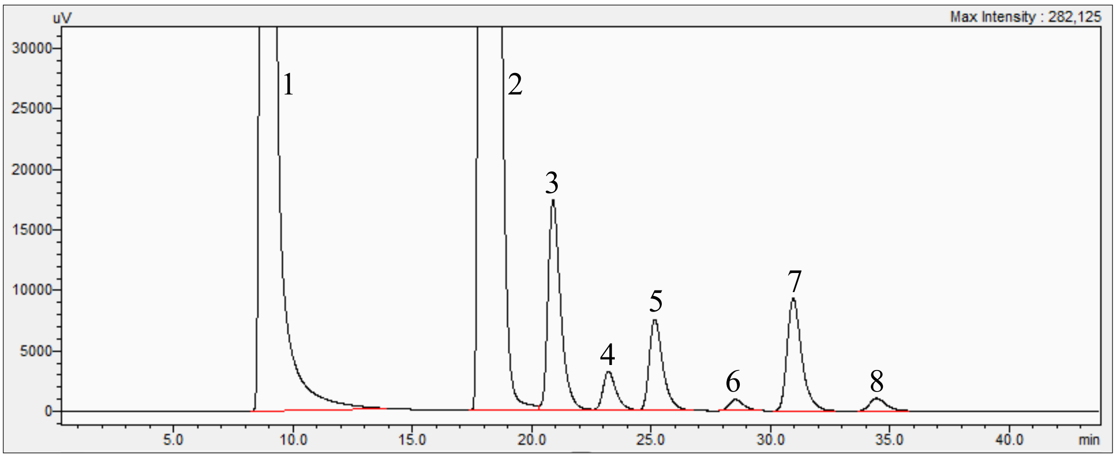

Supplementary Fig. 19 The LC graph of the reaction solution using diethyl ether as substrate.

Entry 13 (dipropyl ether)

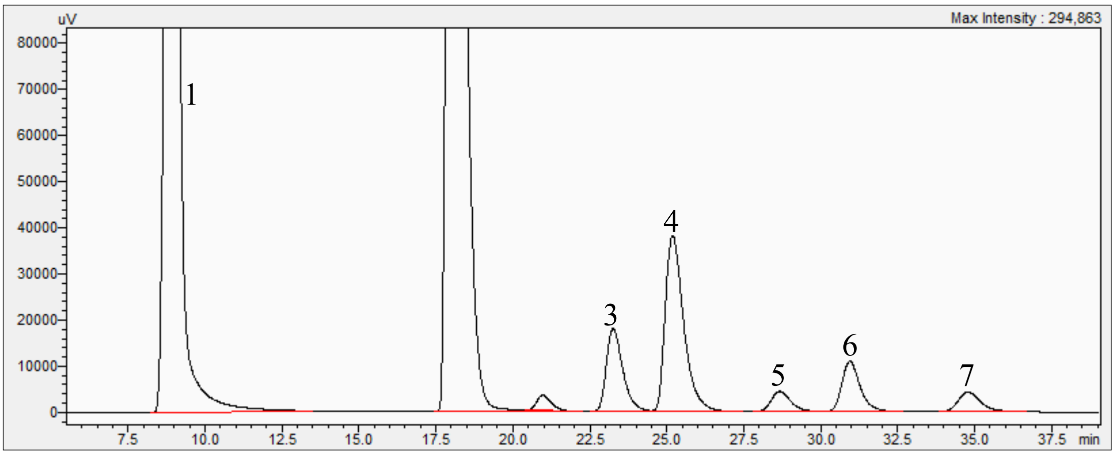

Supplementary Fig. 20 The LC graph of the reaction solution using dipropyl ether as substrate.

Entry 14 (dibutyl ether)

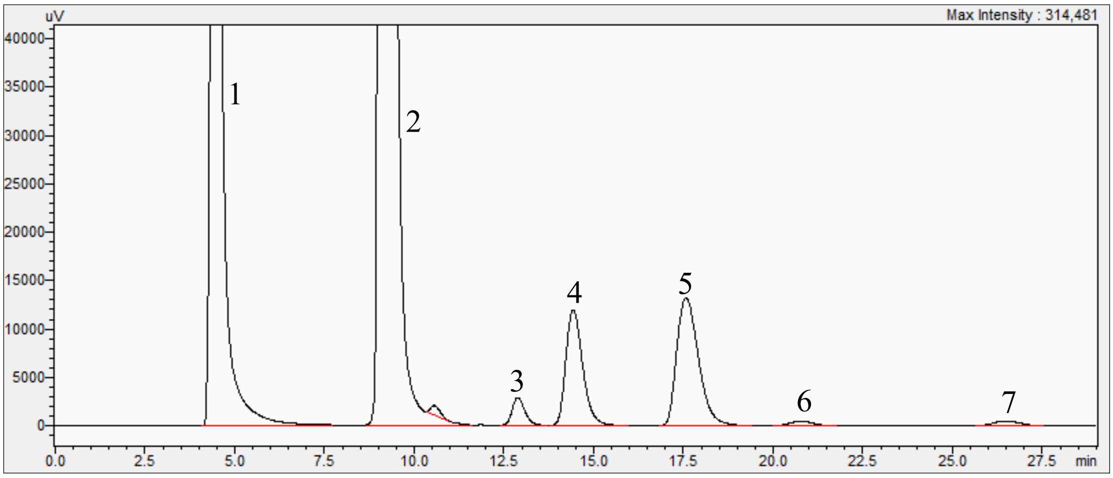

Supplementary Fig. 21 The LC graph of the reaction solution using dibutyl ether as substrate.

Entry 15 (dipentyl ether)

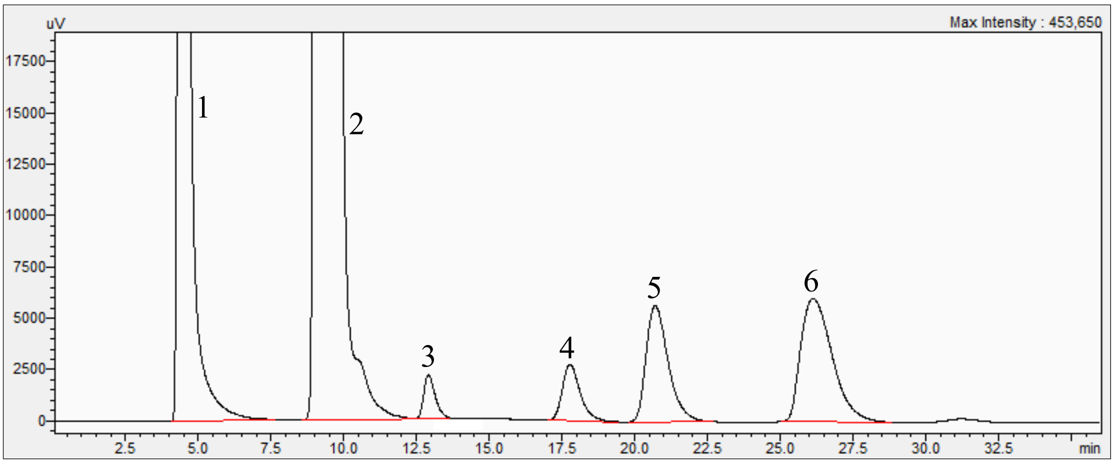

Supplementary Fig. 22 The LC graph of the reaction solution using dipentyl ether as substrate.

Entry 16 (dihexyl ether)

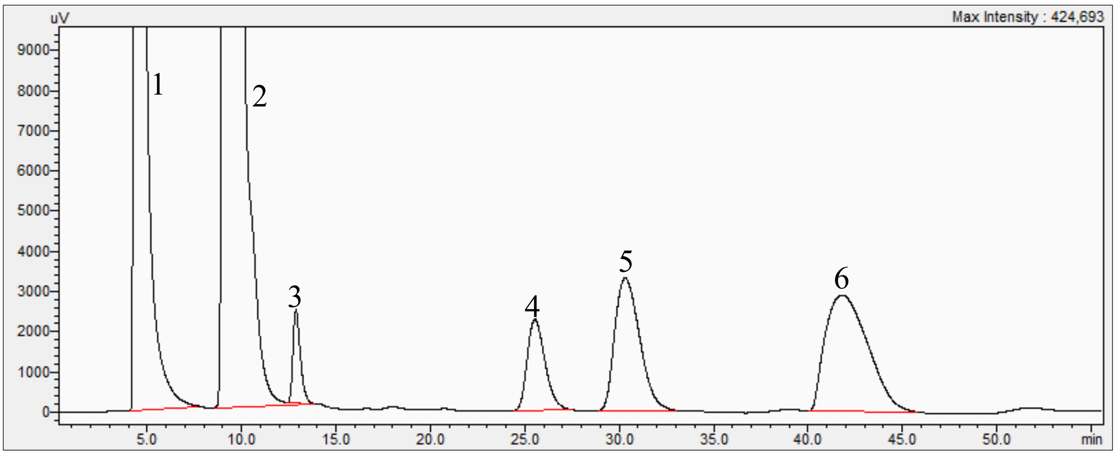

Supplementary Fig. 23 The LC graph of the reaction solution using dihexyl ether as substrate.

Entry 17 (diisopropyl ether)

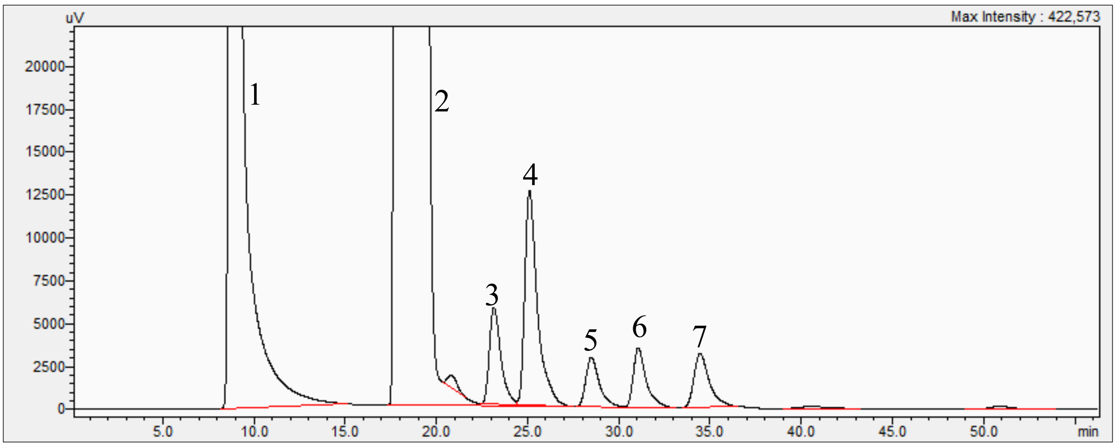

Supplementary Fig. 24 The LC graph of the reaction solution using diisopropyl ether as substrate.

Entry 18 (sec-butyl ether)

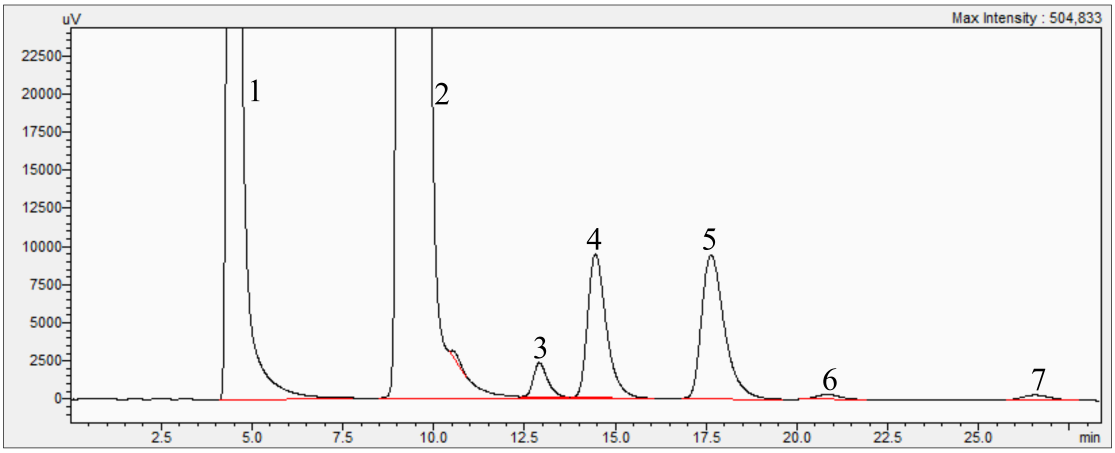

Supplementary Fig. 25 The LC graph of the reaction solution using sec-butyl ether as substrate.

Entry 19 (cyclopentyl methyl ether)

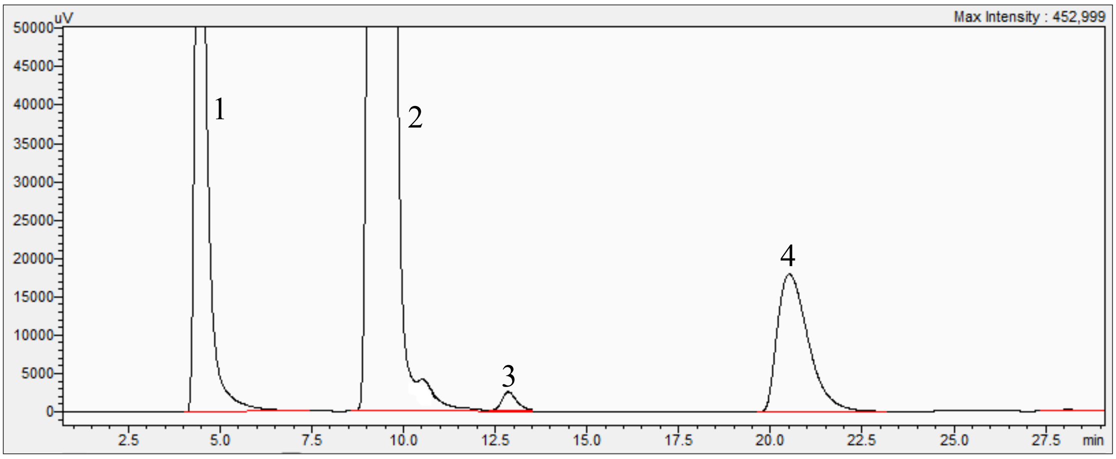

Supplementary Fig. 26 The LC graph of the reaction solution using cyclopentyl methyl ether as substrate.

Entry 20 (phenetole)

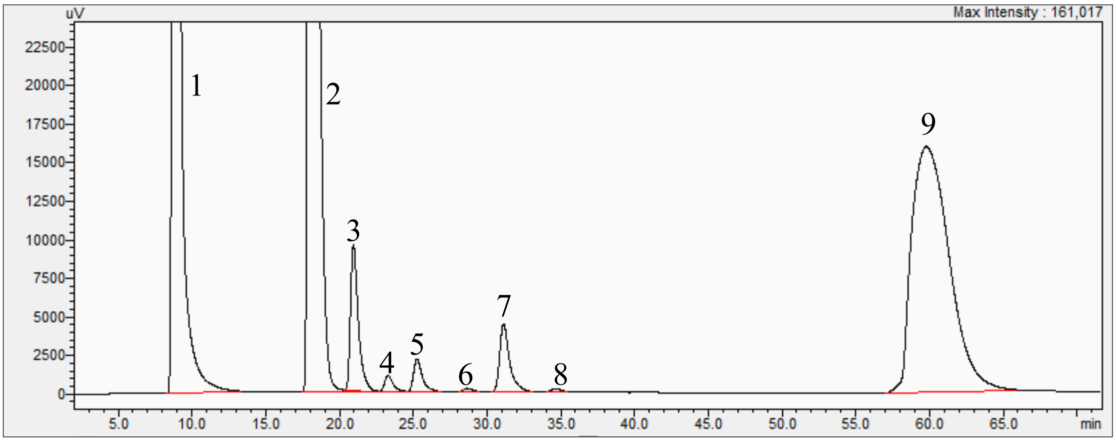

Supplementary Fig. 27 The LC graph of the reaction solution using phenetole as substrate.

Entry 21 (propoxybenzene)

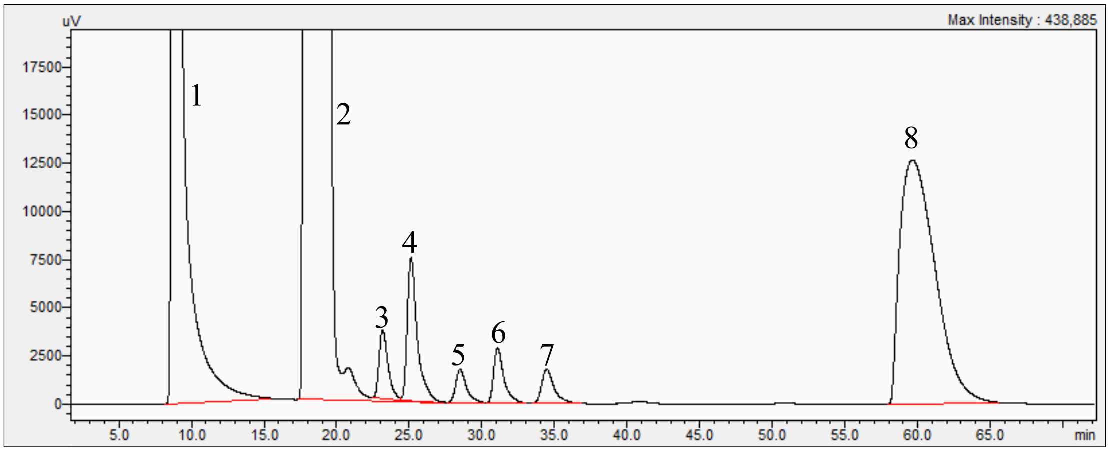

Supplementary Fig. 28 The LC graph of the reaction solution using propoxybenzene as substrate.

Entry 22 (butoxybenzene):

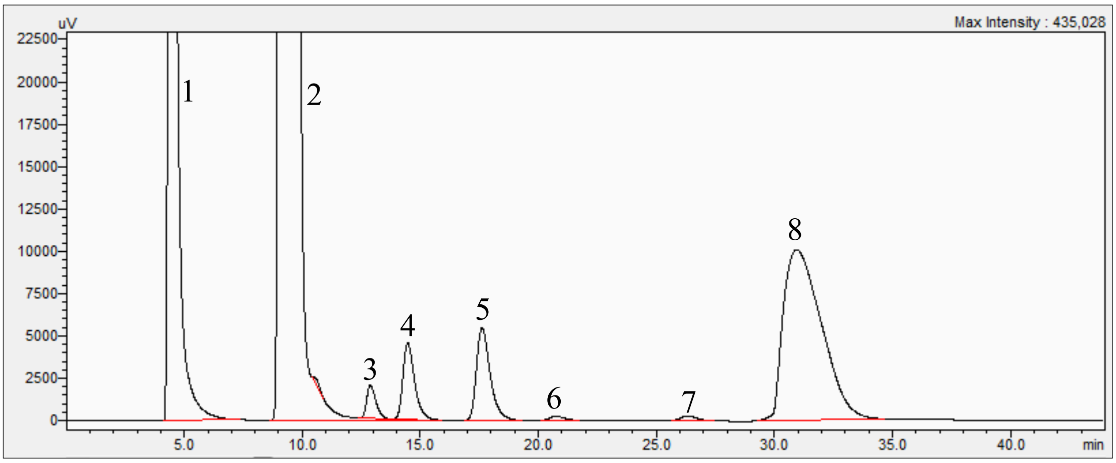

Supplementary Fig. 29 The LC graph of the reaction solution using butoxybenzene as substrate.

Entry 23 (3-butoxyphenol)

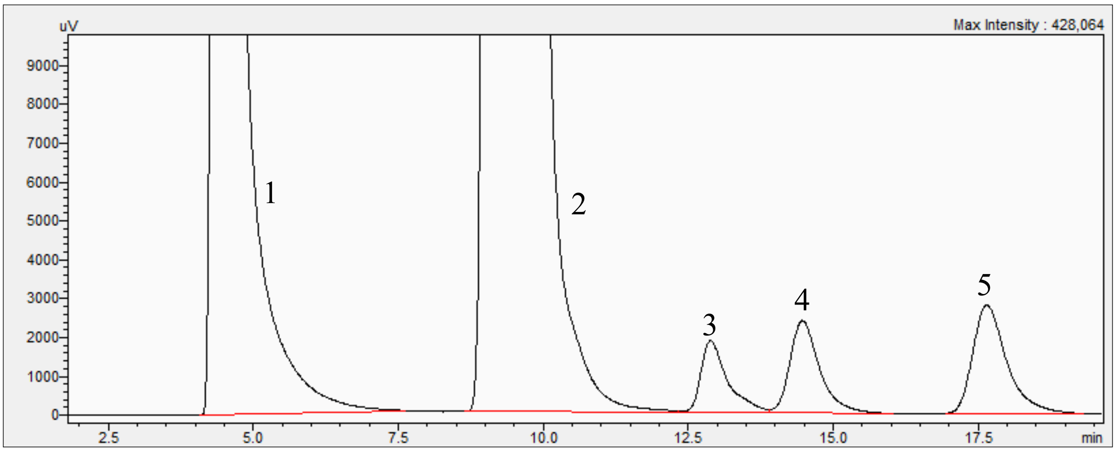

Supplementary Fig. 30 The LC graph of the reaction solution using 3-butoxyphenol as substrate.

Entry 24 (4-butoxyphenol)

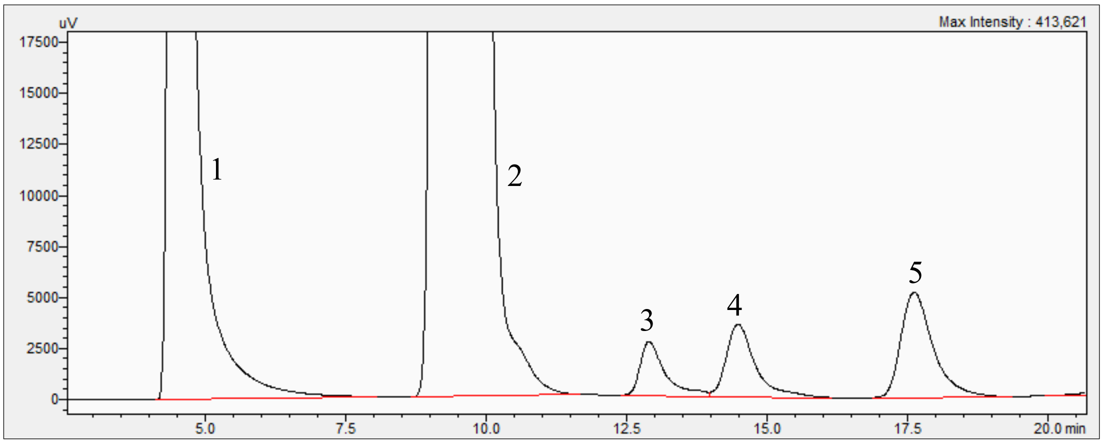

Supplementary Fig. 31 The LC graph of the reaction solution using 4-butoxyphenol as substrate.

Entry 25 (4-butoxyaniline)

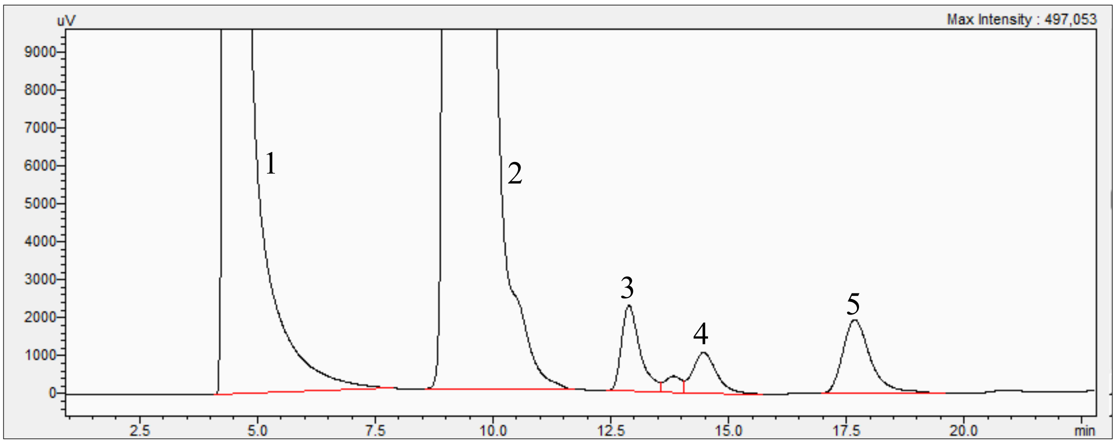

Supplementary Fig. 32 The LC graph of the reaction solution using 4-butoxyaniline as substrate.

Entry 26 (4-butoxybenzoic acid)

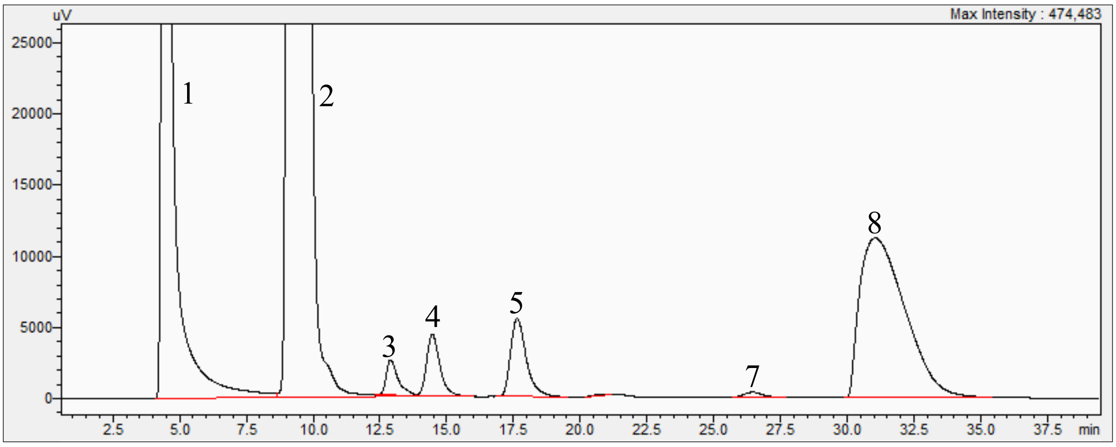

Supplementary Fig. 33 The LC graph of the reaction solution using 4-butoxybenzoic acid as substrate.

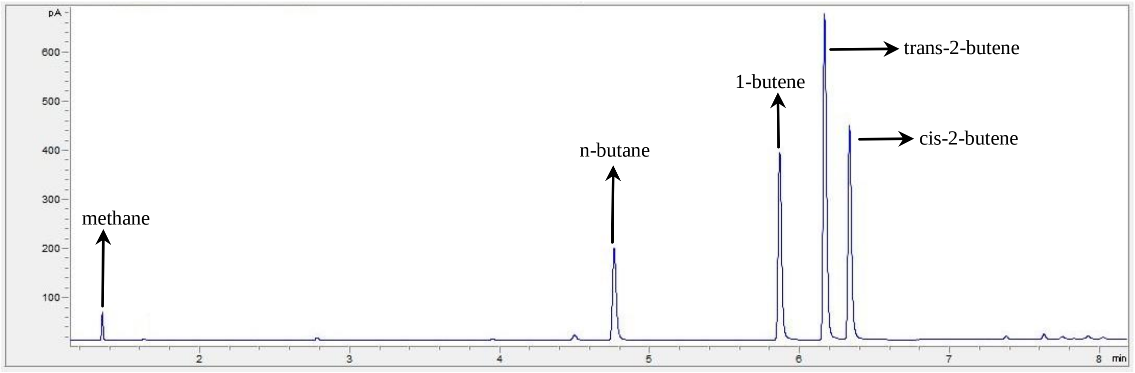

Supplementary Fig. 34 The GC graph of the gaseous sample after 2 h of reaction. Reaction conditions: 20 μmol IrI_4, 2 mmol LiI, 2.45mmol THF, 0.6 mL AcOH, 5 MPa CO_2 (68 mmol) and 2 MPa H_2 (at room temperature), 170 ^oC, 2 h.

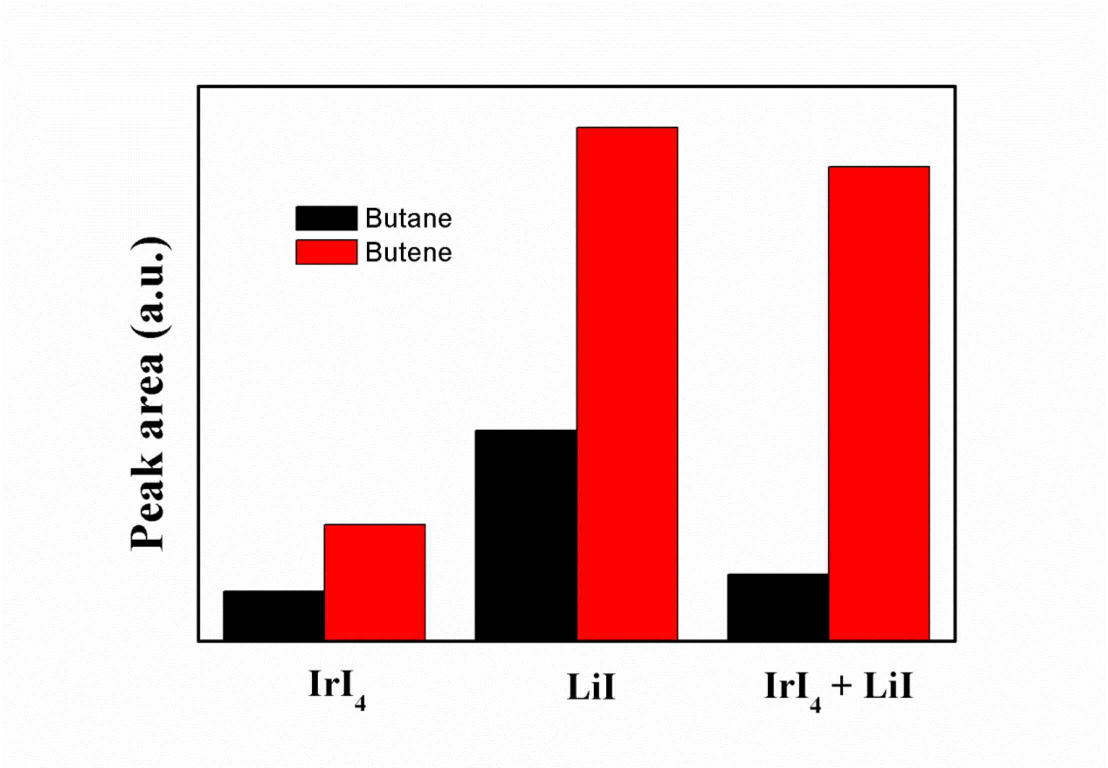

Supplementary Fig. 35 Impact of the catalytic component on generation of butene. Reaction condition: 20 μmol IrI_4 (if used), 2 mmol LiI (if used), 2.45mmol THF, 0.6 mL AcOH, 5 MPa CO_2 (68 mmol) and 2 MPa H_2 (at room temperature), 170 °C, 2 h. The above figure was plotted based on GC data of the gaseous sample. It is very difficult to quantify the amount of each isomeride of butene, and the peak area of the butene was the total peak area of the isomerides.

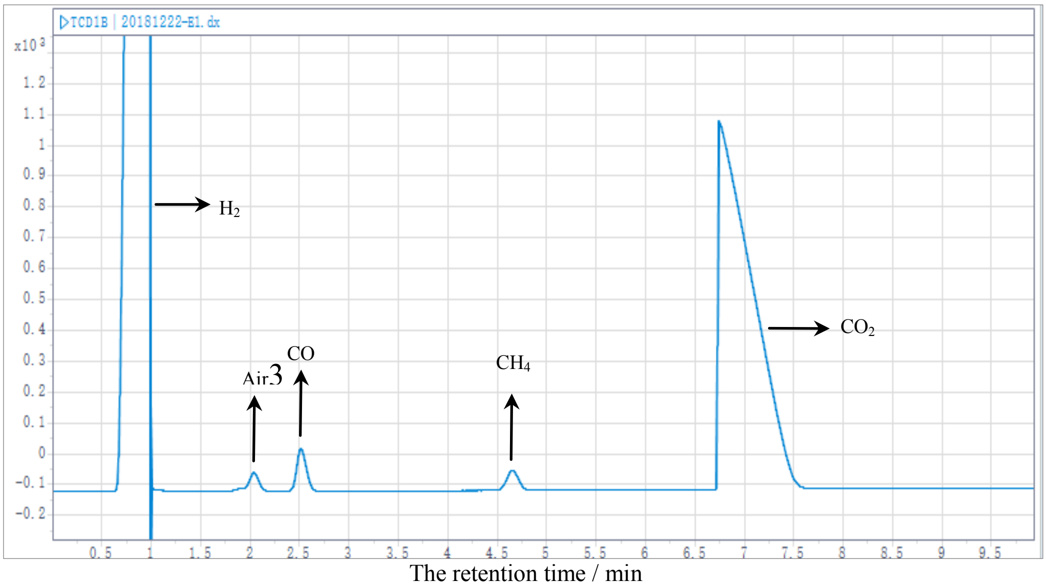

Supplementary Fig. 36 The GC graph of the gaseous sample after RWGS reaction. Reaction conditions: 20 μmol IrI_4, 2 mmol LiI, 0.6 mL AcOH, 5 MPa CO_2 (68 mmol) and 2 MPa H_2 (at room temperature), 170 °C, 16 h.

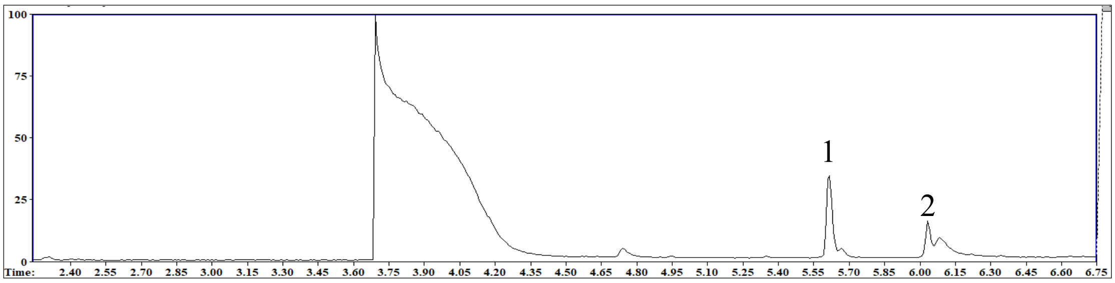

Peak 1:

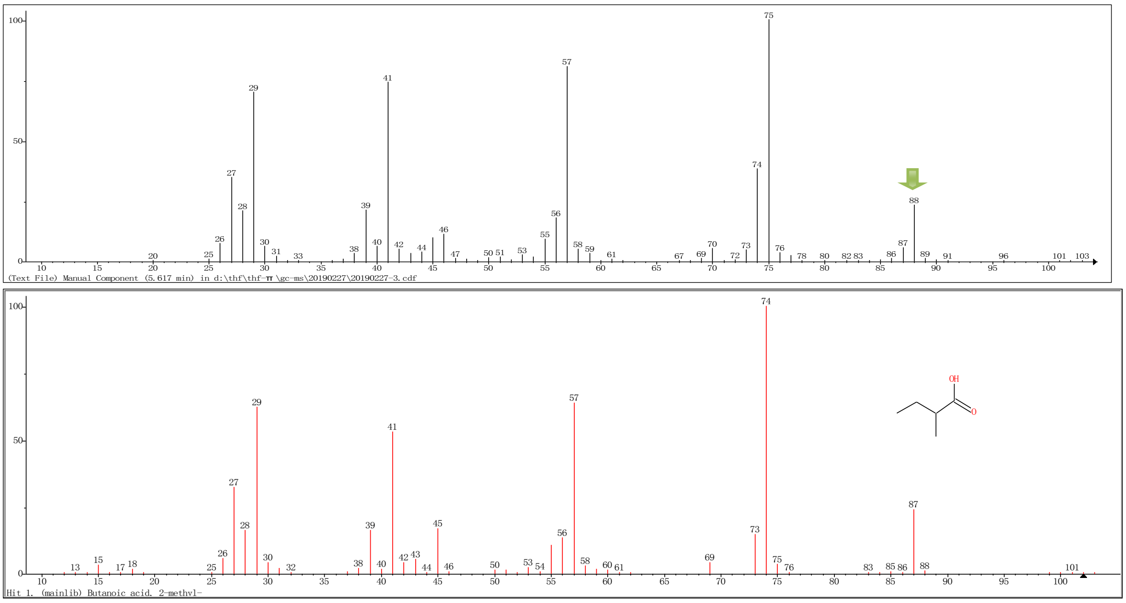

Peak 2:

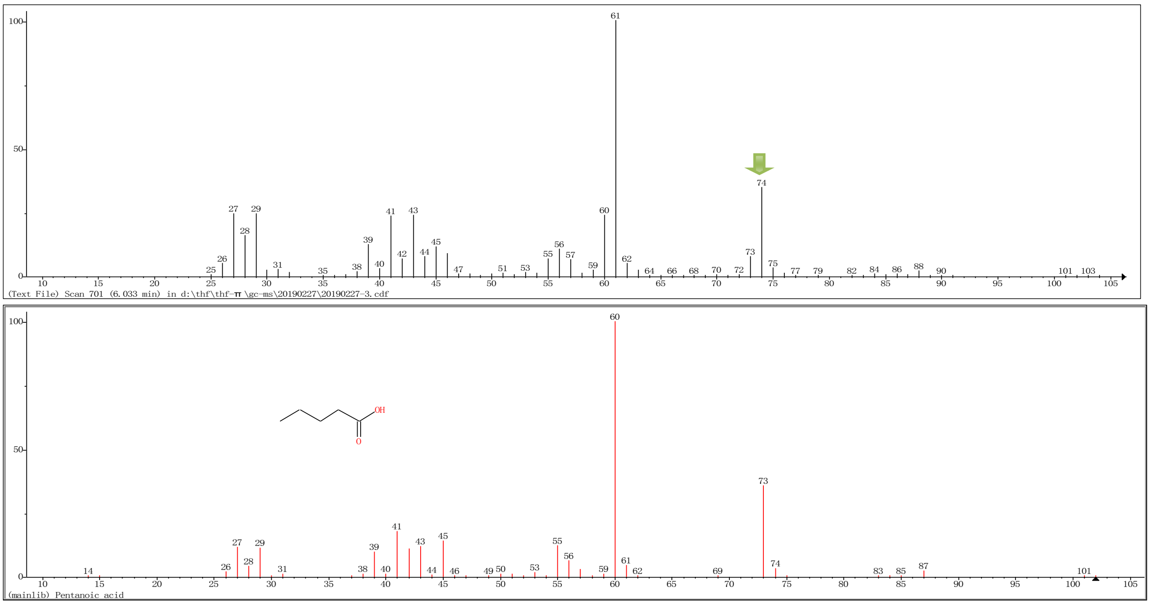

Supplementary Fig. 37 The GC-MS spectra of the $^{13}CO_2$ labeling test. Reaction conditions: 20 μmol IrI_4, 2 mmol LiI, 0.6 mL AcOH, 2.45 mmol THF, 2 MPa $^{13}CO_2$ and 1 MPa H_2 (at room temperature), 170 °C, 16 h.

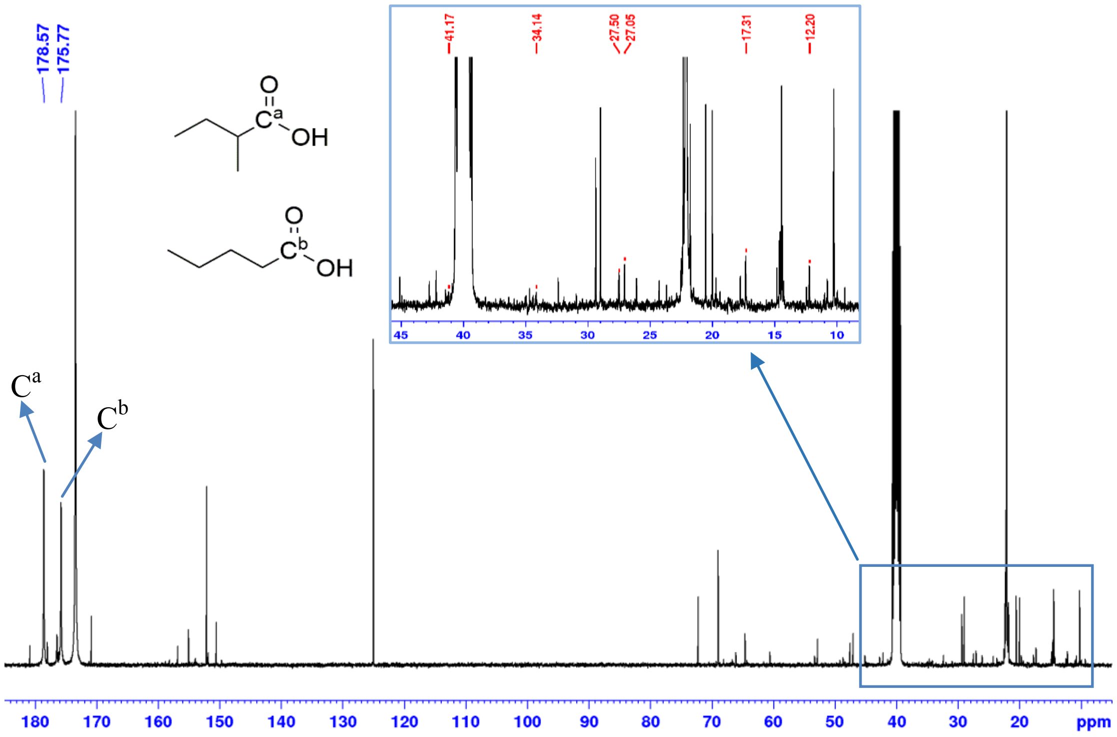

Supplementary Fig. 38 The ^{13}C NMR spectra of $^{13}CO_2$ labeling experiment. Reaction conditions: 20 μmol IrI_4, 2 mmol LiI, 0.6 mL AcOH, 2.45 mmol THF, 2 MPa $^{13}CO_2$ and 1 MPa H_2 (at room temperature), 170 ^{o}C, 16 h. The small peaks (δ 41.17 ppm, 34.14 ppm, 27.50 ppm, 27.05 ppm, 17.31 ppm, 12.20 ppm) belong to alkyl carbons of pentanoic acid and 2-methylbutanoic acid products. The peaks of C^a and C^b are attributed to the carbons of the carboxyl groups in the products, which are remarkably larger than those of the alkyl carbons of the products. This indicated that CO_2 took part in forming the carboxyl group of the products.

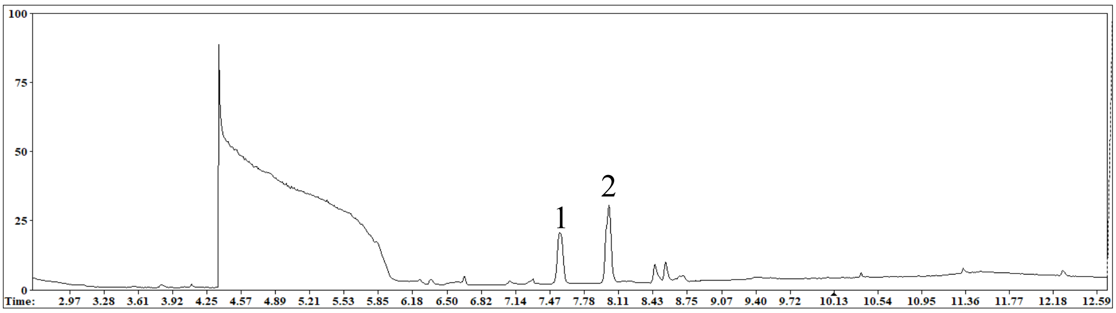

Peak 1：

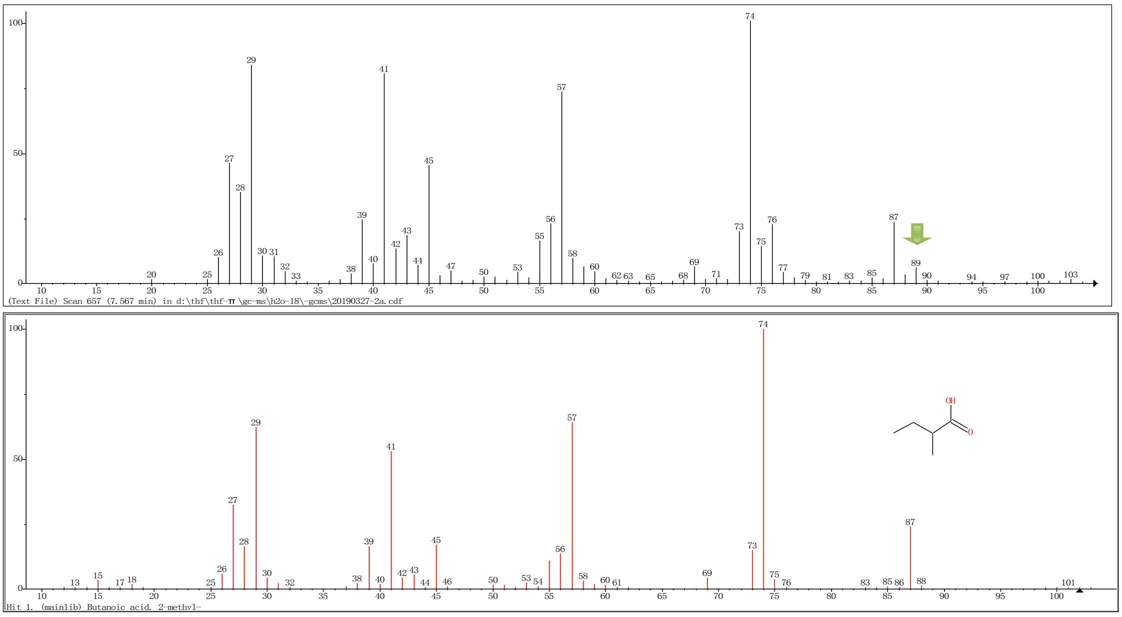

Peak 2：

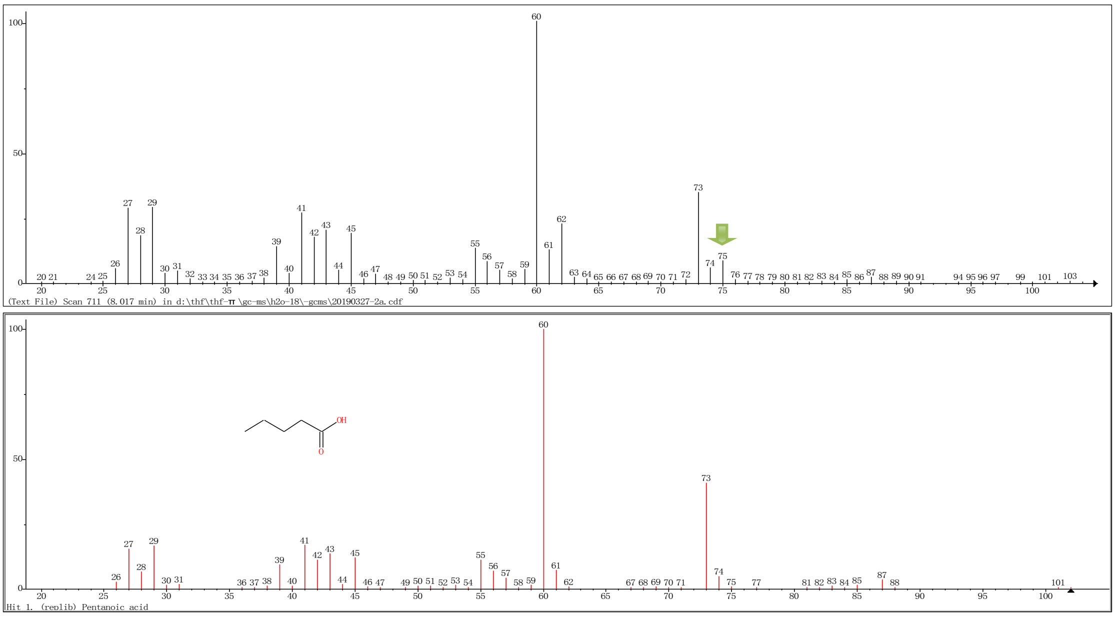

Supplementary Fig. 39 The GC-MS spectra of $H_2{}^{18}O$ labeling experiment. Reaction conditions: 20 μmol IrI_4, 2 mmol LiI, 0.3 mL $H_2{}^{18}O$, 0.6 mL AcOH, 2.45 mmol THF, 5 MPa CO_2 (68 mmol) and 2 MPa H_2 (at room temperature), 170 ^oC, 16 h. The result suggested that OH in COOH group was from H_2O generated in situ.

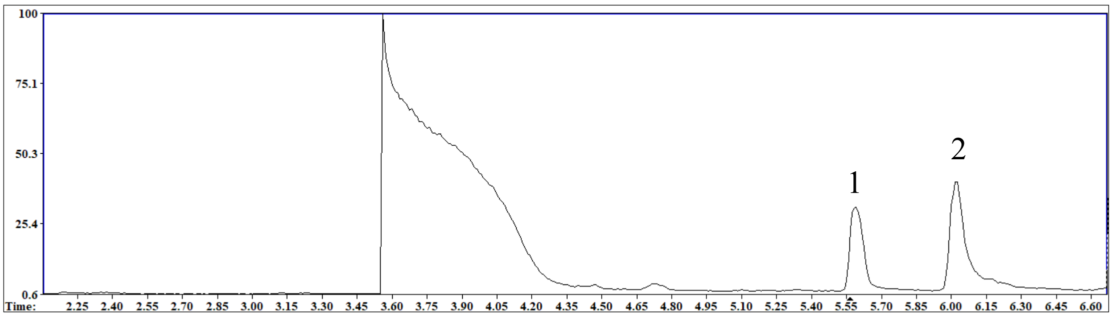

Peak 1:

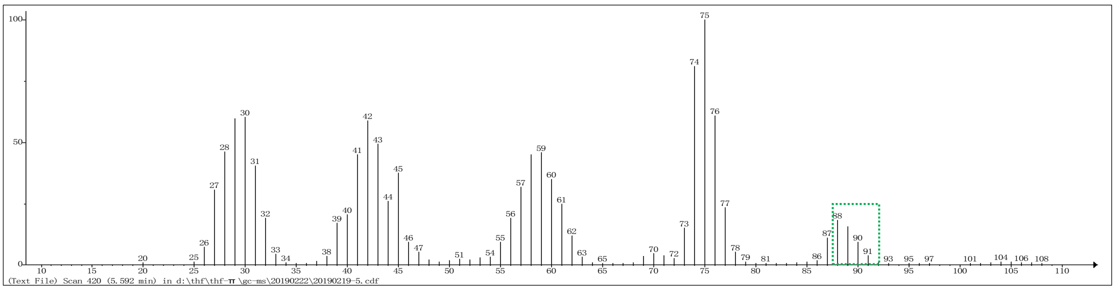

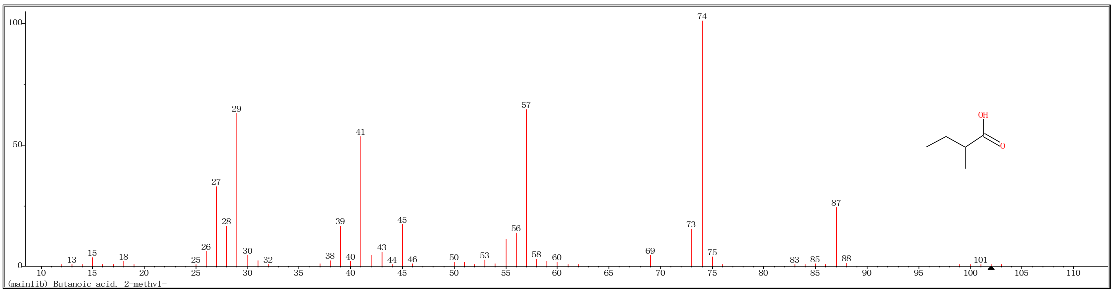

Peak 2:

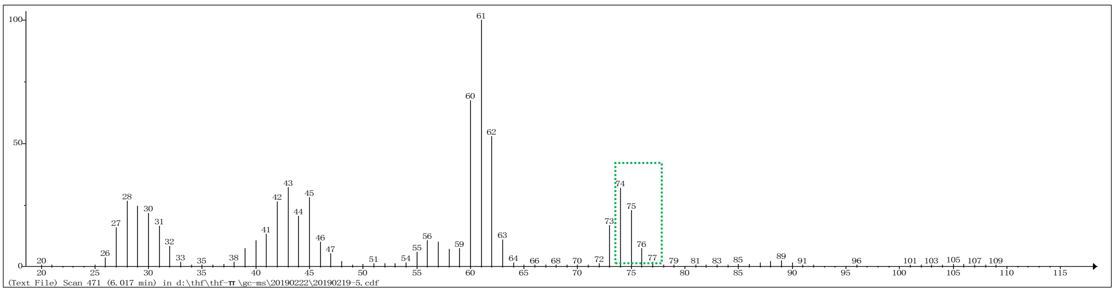

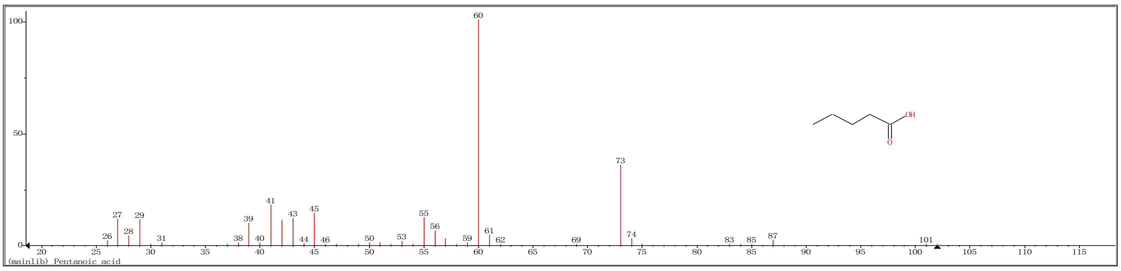

Supplementary Fig. 40 The GC-MS spectra of D_2 labelling experiments. Reaction conditions: 20 μmol IrI_4, 2 mmol LiI, 0.6 mL AcOH, 2.45 mmol THF, 5 MPa CO_2 (68 mmol) and 2 MPa D_2 (at room temperature), 170 ^{o}C, 16 h.

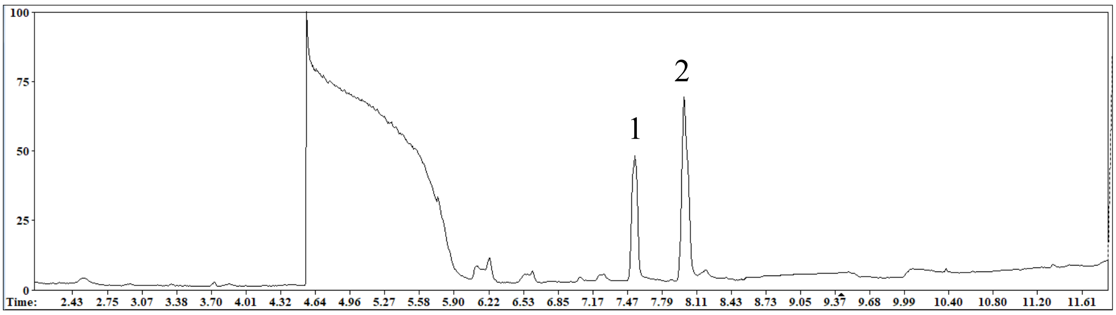

Peak 1:

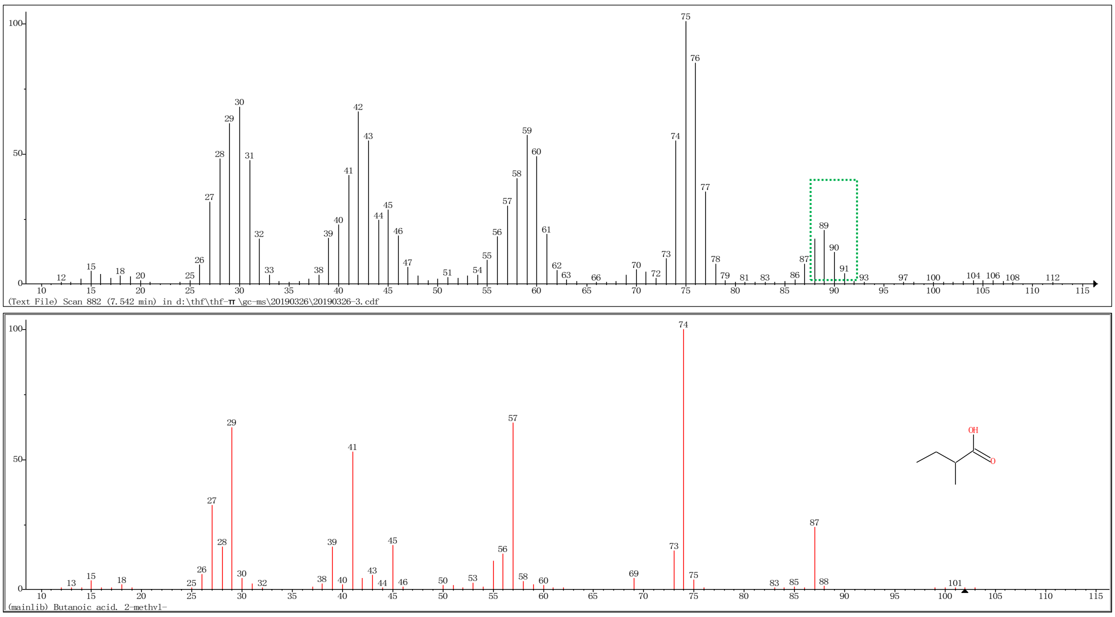

Peak 2:

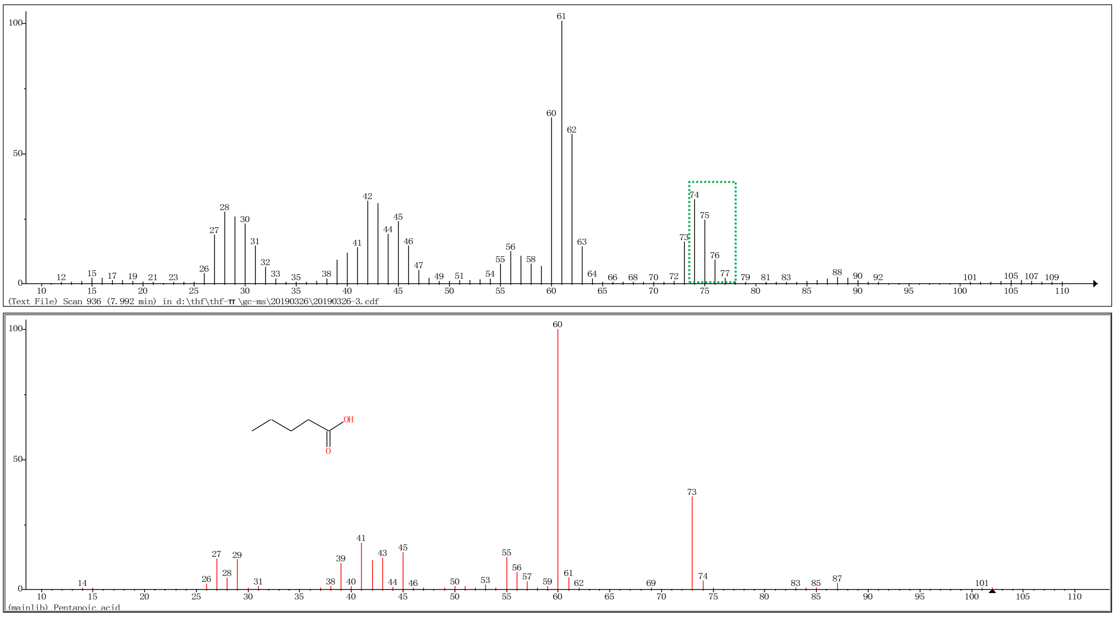

Supplementary Fig. 41 The GC-MS spectra of D_2O labeling experiment. Reaction conditions: 20 μmol IrI_4, 2 mmol LiI, 0.3 mL D_2O, 0.6 mL AcOH, 2.45 mmol THF, 5 MPa CO_2 (68 mmol) and 2 MPa H_2 (at room temperature), 170 ^{o}C, 16 h.

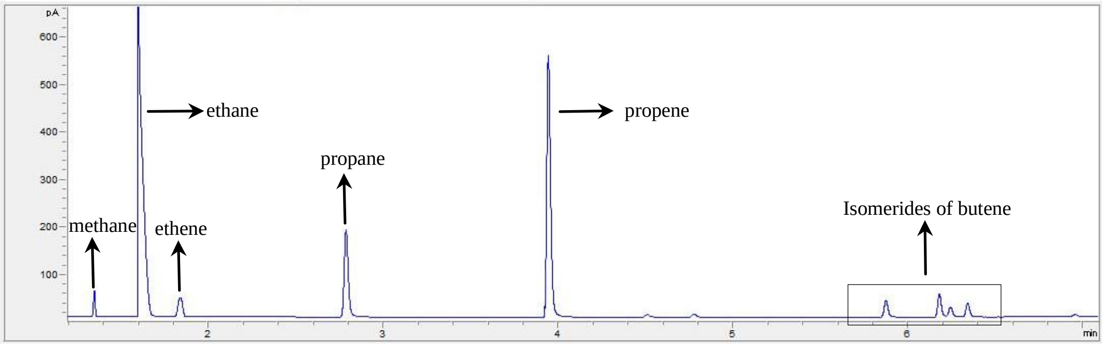

Supplementary Fig. 42 The GC graph of the gaseous sample after 2 h reaction of diethyl ether, CO_2 and H_2. Reaction conditions: 20 μmol IrI_4, 2 mmol LiI, 1.23 mmol diethyl ether, 0.6 mL AcOH, 5 MPa CO_2 (68 mmol) and 2 MPa H_2 (at room temperature), 170 ^oC, 2 h.

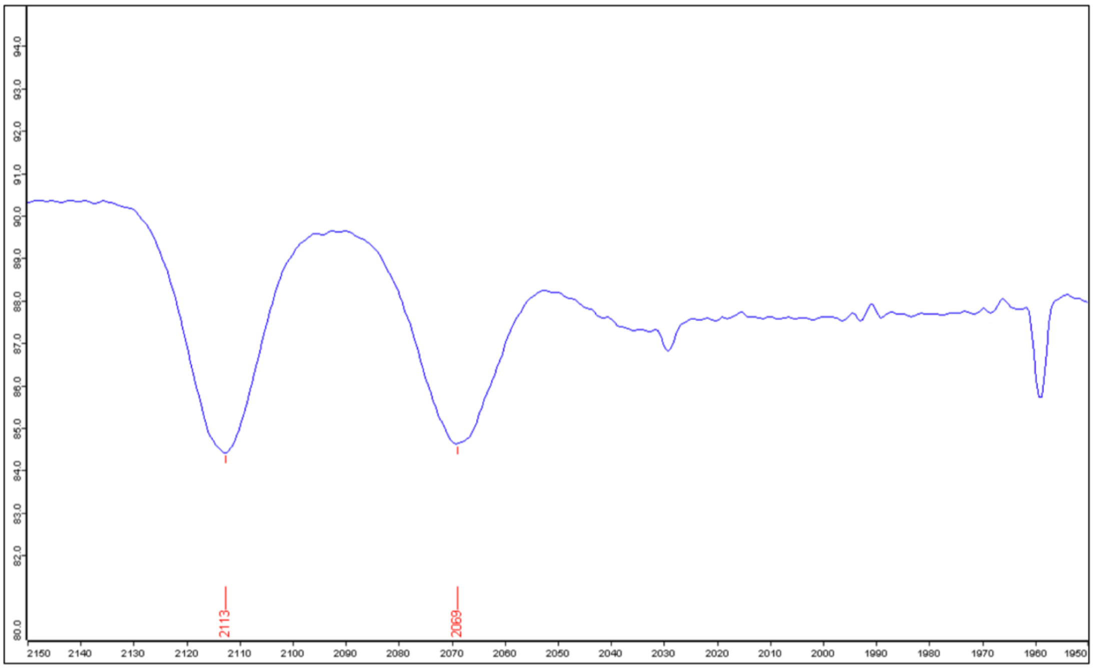

Supplementary Fig. 43 IR spectra of the liquid sample after the reaction. Reaction conditions: 20 μmol IrI_4, 2 mmol LiI, 0.6 mL AcOH, 2.45 mmol THF, 5 MPa CO_2 (68 mmol) and 2 MPa H_2 (at room temperature), 170 ^{o}C, 16 h. According to reference, ν (CO) bands appeared at 2113 and 2069 cm^{-1} demonstrate the presence of cis-$[Ir(CO)_2I_4]^-$ species[1].

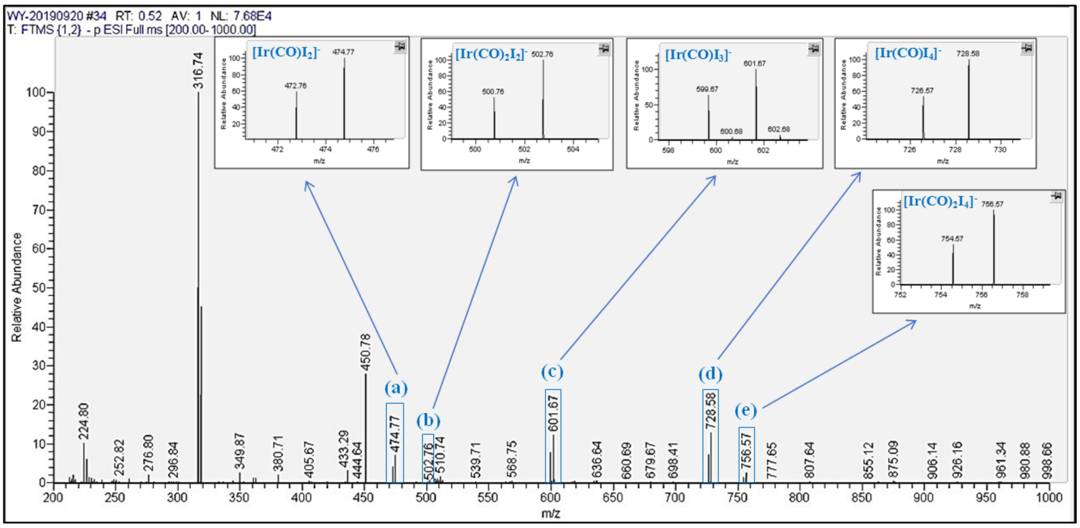

Supplementary Fig. 44 HR-ESI(-)-MS of the liquid sample after the reaction. Reaction conditions: 20 μmol IrI_4, 2 mmol LiI, 0.6 mL AcOH, 2.45 mmol THF, 5 MPa CO_2 (68 mmol) and 2 MPa H_2 (at room temperature), 170 ^oC, 16 h. The spectra of detected Ir species are in good agreement with their calculated isotope distribution spectra.

Supplementary Tables

Supplementary Table 1 The price of some substrates, products and metals.

Substrates	Products
propyl diisoether, 80 ¥/L (Aladdin)	2-methylpropionic acid, 203 ¥/kg (Aldrich)
	butyric acid, 207 ¥/L (Aldrich)
THF, 78 ¥/L (Aladdin)	2-methylbutyric acid, 560 ¥/kg (Aldrich)
or dibutyl ether, 56 ¥/L (Aladdin)	valeric acid, 246 ¥/kg (Aldrich)
2-methyltetrahydrofuran, 400 ¥/L (Aladdin)	2-ethylbutyric acid, 2014 ¥/kg (Aldrich)
or Tetrahydropyran, 690 ¥/L (Aladdin)	2-methylvaleric acid, 2377 ¥/kg (Aldrich)
dihexyl ether, 7128 ¥/L (Aladdin)	2-ethylvaleric acid, 6528300 ¥/kg (Key Oranics)
	2-methylhexanoic acid, 11868 ¥/kg (Aldrich)
**Metals**	
Iridium, 1055 ¥/g (Alfa)	Rhodium, 10727 ¥/g (Alfa)

The above price was obtained from the following website in June, 2019. Sigma- Aldrich Co, LLC. https://www.sigmaaldrich.com/china-mainland.html; Alfa Aesar. https://www.alfa.com/zh-cn; Aladdin. https://www.aladdin-e.com/zh_cn; Key Oranics Limited. https://www.keyorganics.net

Supplementary Table 2 The performance of different Ir catalyst precursors.

Entry	Catalyst precursor	Promoter	Solvent	Yield [%][§]
1	IrI_4	LiI	AcOH	70
2	$Ir(CO)(PPh_3)_2Cl$	LiI	AcOH	17
3	$Ir(CO)_2(acac)$	LiI	AcOH	63
4	$IrCl_3$	LiI	AcOH	64

Reaction conditions: 20 μmol catalyst precursor, 2 mmol LiI, 0.6 mL AcOH, 2.45 mmol THF, 5 MPa CO_2 (68 mmol) and 2 MPa H_2 (at room temperature), 170 ^{o}C, 16 h. [§]Yield is based on THF feedstock (100 × moles of C_5 carboxylic acids per mole of THF feedstock).

Supplementary Table 3 Impact of different acids on the reaction using THF as solvent and reactant.

Entry	Acid	(2a+2a') [mmol]	Selectivity of (2a+2a') [%]	2a/(2a+2a') [%]
1	AcOH	0.29	4.6	76
2	CF_3COOH	0.37	4.3	76
3	CH_3SO_3H	0.35	4.0	74
4	H_3PO_4	<0.01	-	-
5	$Al(OTf)_3$	<0.01	-	-

Reaction conditions: 20 μmol IrI_4, 2 mmol LiI, 1.54 mmol acid, 0.7 mL THF, 5 MPa CO_2 (68 mmol) and 2 MPa H_2 (at room temperature), 170 ^oC, 8 h.

Supplementary Table 4 Effect of reaction parameters on synthesizing carboxylic acids from THF, CO_2 and H_2.

Entry	IrI_4 [μmol]	LiI [mmol]	CO_2/H_2 [MPa]	Yield [%] of (2a+2a')
1	10	2	5/2	45
2	20	2	5/2	70
3	30	2	5/2	68
4	40	2	5/2	65
5	20	1	5/2	29
6	20	3	5/2	62
7	20	4	5/2	45
8	20	2	0/0	0
9	20	2	5/0	0
10	20	2	0/2	0
11	20	2	6/1	41
12	20	2	3.5/3.5	27
13	20	2	2.3/4.7	23
14	20	2	2.5/1	12
15	20	2	6/2.4	71
16†	20	2	5/2	0

Reaction conditions: IrI_4 was used as the catalyst, LiI was used as the promoter, 0.6 mL acetic acid, 2.45 mmol THF, 170 ^{o}C, 16 h. †No THF was added in the reaction.

Supplementary Table 5 Catalytic results using iodides/acetates to react with CO.

Entry	Catalyst	Promoter	Substrate	Product	Yield [%]	Products Distribution
1	IrI_4	-	1-iodobutane		72	1 : 2 = 57 : 43
2[#]	$Ir_4(CO)_{12}$	-	1-iodobutane		55	1 : 2 = 65 : 35
3	IrI_4	LiI	1-iodobutane	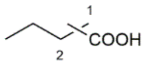	74	1 : 2 = 41 : 59
4	IrI_4	-	1-butyl acetate		12	1 : 2 = 76 : 24
5[#]	$Ir_4(CO)_{12}$	-	1-butyl acetate		< 1	-
6	IrI_4	LiI	1-butyl acetate		84	1 : 2 = 45 : 55
7	IrI_4	-	cyclohexene		35	-
8[#]	$Ir_4(CO)_{12}$	-	cyclohexene	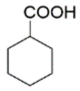	< 1	-
9	IrI_4	LiI	cyclohexene		53	-

Reaction conditions: 20 μmol Ir catalyst (based on the metal), 2 mmol LiI, 0.6 mL AcOH, 0.5 mmol substrate, 1 MPa CO (at room temperature), 170 °C, 8 h. [#] $Ir_4(CO)_{12}$ was used instead of IrI_4 to exclude the influence of iodide in the IrI_4 on the reaction.

Supplementary Table 6 Catalytic results using iodides/acetates to react with CO_2 and H_2.

Entry	Substrate	Yield [%] of (2a+2a')	2a/(2a+2a') [%]
1	1-iodobutane	17	55
2	2-iodobutane	15	54
3	1-butyl acetate	60	56
4	2-butyl acetate	44	54

Reaction condition: 20 μmol IrI_4, 2 mmol LiI, 0.6 mL acetic acid, 2.45 mmol substrate, 5 MPa CO_2 (68 mmol) and 2 MPa H_2 (at room temperature), 170 ^oC, 16 h. The alkyl iodides were easy to be converted to corresponding alkanes in the presence of H_2. The alkyl iodides were more easily transformed into alkanes compared to reacting with CO generated via RWGS reaction, which may explain why the yields of entries 1 and 2 were much lower.

Supplementary Table 7 Control experiments using CO instead of CO_2

Entry	CO [MPa]	H_2 [MPa]	Yield [%] of (2a+2a')	2a/(2a+2a') [%]
1	1	0	62	53
2	0.5	2	64	66
3	1	2	67	57
4	3	2	39	61

Reaction conditions: 20 μmol IrI_4, 2 mmol LiI, 0.6 mL acetic acid, 2.45 mmol THF, 170 ^oC, 16 h. Minor α-methyl butyrolactone and γ-methyl butyrolactone were observed without H_2 (entry 1), while they disappeared when H_2 was charged before the reaction (entries 2-4).

Supplementary Table 8 Catalytic performance of heterogeneous Ir catalysts.

Entry	Catalyst	Promoter	Solvent	Yield [%] [§]
1 [#]	Ir	LiI	AcOH	0
2	Ir	LiI	AcOH	0
3	Ir/AC	LiI	AcOH	3

Reaction conditions: 20 μmol Ir catalyst (based on the metal), 2 mmol LiI, 0.6 mL AcOH, 2.45 mmol THF, 5 MPa CO_2 (68 mmol) and 2 MPa H_2 (at room temperature), 170 ^{o}C, 16 h. [§]Yield is based on THF feedstock (100 × moles of C_5 carboxylic acids per mole of THF feedstock). [#] Iridium powder (325 mesh, 99.9% (metals basis)) was purchased from Alfa Aesar company. The procedures to prepare the catalysts in entries 2 and 3 were given in the Supplementary methods.

Supplementary Table 9 The retention time of the chemicals in the LC traces.

Entry	Retention time [min]	Substance	The flow rate of mobile phase [mL/min]
1	8.85-8.95	LiI (promoter)	0.5 mL/min
2	18.05-18.75	AcOH (solv.)	
3	20.80-21.00	propionic acid	
4	23.15-23.30	2-methylpropionic acid	
5	25.10-25.30	butyric acid	
6	28.45-28.70	2-methylbutyric acid	
7	30.90-31.15	DMSO (stand.)	
8	34.45-34.80	valeric acid	
9	4.40-4.50	LiI (promoter)	1.0 mL/min
10	9.15-9.90	AcOH (solv.)	
11	10.55-10.65	propionic acid	
12	12.85-12.95	dioxane (stand.)	
13	14.40-14.50	2-methylbutyric acid	
14	17.55-17.70	valeric acid	
15	17.75-17.80	2-ethylbutyric acid	
16	20.50-20.55	cyclopentanecarboxylic acid	
17	20.70-20.90	2-methylvaleric acid	
18	25.25-25.65	2-ethylvaleric acid	
19	26.10-26.55	hexanoic acid	
20	28.80-28.90	cyclohexanecarboxylic acid	
21	29.80-30.45	2-methylhexanoic acid	
22	40.70-42.05	heptanoic acid	

The column temperature was maintained at 323.15 K. The column was eluted with 5 mmol/L H_2SO_4 solution at a flow rate of 0.5 mL/min or 1.0 mL/min according to the samples analyzed. In addition, solvent (solv.) and internal standard (stand.) were also marked in the graphs.

Supplementary Table 10 The peak information in Supplementary Fig. 8.

Peak	Retention time [min]	Substance	Area
1	4.470	LiI (promoter)	2100545
2	9.436	AcOH (solv.)	15064079
3	12.895	dioxane (stand.)	68579
4	14.429	2-methylbutyric acid	384603
5	17.552	valeric acid	526640
6	20.751	2-methylvaleric acid	17367
7	26.352	hexanoic acid	24891

The flow rate of mobile phase was maintained at 1.0 mL/min.

Supplementary Table 11 The peak information in Supplementary Fig. 9.

Peak	Retention time [min]	Substance	Area
1	4.454	LiI (promoter)	1327338
2	9.262	AcOH (solv.)	9587399
3	12.889	dioxane (stand.)	41751
4	17.757	2-ethylbutyric acid	61079
5	20.711	2-methylvaleric acid	152022
6	26.162	hexanoic acid	238011

The flow rate of mobile phase was maintained at 1.0 mL/min.

Supplementary Table 12 The peak information in Supplementary Fig. 10.

Peak	Retention time [min]	Substance	Area
1	4.453	LiI (promoter)	2365463
2	9.646	AcOH (solv.)	16716382
3	12.887	dioxane (stand.)	66774
4	25.625	2-ethylvaleric acid	139036
5	30.446	2-methylhexanoic acid	225474
6	42.018	heptanoic acid	354697

The flow rate of mobile phase was maintained at 1.0 mL/min.

Supplementary Table 13 The peak information in Supplementary Fig. 11.

Peak	Retention time [min]	Substance	Area
1	4.458	LiI (promoter)	1010388
2	9.251	AcOH (solv.)	8324099
3	12.886	dioxane (stand.)	33903
4	14.500	2-methylbutyric acid	56688
5	17.671	valeric acid	88892

The flow rate of mobile phase was maintained at 1.0 mL/min.

Supplementary Table 14 The peak information in Supplementary Fig. 12.

Peak	Retention time [min]	Substance	Area
1	8.925	LiI (promoter)	8032937
2	18.143	AcOH (solv.)	9539768
3	23.231	2-methylpropionic acid	750353
4	25.141	butyric acid	1519421
5	28.582	2-methylbutyric acid	231864
6	30.977	DMSO (stand.)	473177
7	34.542	valeric acid	235283

The flow rate of mobile phase was maintained at 0.5 mL/min.

Supplementary Table 15 The peak information in Supplementary Fig. 13.

Peak	Retention time [min]	Substance	Area
1	4.450	LiI (promoter)	2019690
2	9.427	AcOH (solv.)	13702999
3	12.899	dioxane (stand.)	69799
4	17.797	2-ethylbutyric acid	75779
5	20.724	2-methylvaleric acid	249781
6	26.138	hexanoic acid	405600

The flow rate of mobile phase was maintained at 1.0 mL/min.

Supplementary Table 16 The peak information in Supplementary Fig. 14.

Peak	Retention time [min]	Substance	Area
1	4.427	LiI (promoter)	4601830
2	9.851	AcOH (solv.)	25780300
3	12.853	dioxane (stand.)	164993
4	25.250	2-ethylvaleric acid	285827
5	29.825	2-methylhexanoic acid	568852
6	40.737	heptanoic acid	863629

The flow rate of mobile phase was maintained at 1.0 mL/min.

Supplementary Table 17 The peak information in Supplementary Fig. 15.

Peak	Retention time [min]	Substance	Area
1	8.876	LiI (promoter)	4066617
2	18.682	AcOH (solv.)	30424490
3	23.163	2-methylpropionic acid	261604
4	25.111	butyric acid	653299
5	28.508	2-methylbutyric acid	70355
6	31.078	DMSO (stand.)	191744
7	34.501	valeric acid	80339

The flow rate of mobile phase was maintained at 0.5 mL/min.

Supplementary Table 18 The peak information in Supplementary Fig. 16.

Peak	Retention time [min]	Substance	Area
1	4.439	LiI (promoter)	2226713
2	9.371	AcOH (solv.)	13943198
3	12.857	dioxane (stand.)	60868
4	20.543	cyclopentanecarboxylic acid	866320

The flow rate of mobile phase was maintained at 1.0 mL/min.

Supplementary Table 19 The peak information in Supplementary Fig. 17.

Peak	Retention time [min]	Substance	Area
1	4.457	LiI (promoter)	2444248
2	9.400	AcOH (solv.)	11668207
3	12.855	dioxane (stand.)	72580
4	28.809	cyclohexanecarboxylic acid	1099136

The flow rate of mobile phase was maintained at 1.0 mL/min.

Supplementary Table 20 The peak information in Supplementary Fig. 18.

Peak	Retention time [min]	Substance	Area
1	8.890	LiI (promoter)	4811286
2	18.101	AcOH (solv.)	5277434
3	20.921	propionic acid	432631
4	23.248	2-methylpropionic acid	77811
5	25.192	butyric acid	224503
6	28.579	2-methylbutyric acid	11531
7	31.132	DMSO (stand.)	212671
8	34.476	valeric acid	11490

The flow rate of mobile phase was maintained at 0.5 mL/min.

Supplementary Table 21 The peak information in Supplementary Fig. 19.

Peak	Retention time [min]	Substance	Area
1	8.912	LiI (promoter)	8235749
2	18.088	AcOH (solv.)	9418173
3	20.902	propionic acid	645155
4	23.220	2-methylpropionic acid	116973
5	25.164	butyric acid	295819
6	28.539	2-methylbutyric acid	38334
7	30.963	DMSO (stand.)	394284
8	34.468	valeric acid	50453

The flow rate of mobile phase was maintained at 0.5 mL/min.

Supplementary Table 22 The peak information in Supplementary Fig. 20.

Peak	Retention time [min]	Substance	Area
1	8.919	LiI (promoter)	8119616
2	18.077	AcOH (solv.)	8950132
3	23.255	2-methylpropionic acid	667723
4	25.190	butyric acid	1597588
5	28.666	2-methylbutyric acid	193074
6	30.943	DMSO (stand.)	452392
7	34.779	valeric acid	218563

The flow rate of mobile phase was maintained at 0.5 mL/min.

Supplementary Table 23 The peak information in Supplementary Fig. 21.

Peak	Retention time [min]	Substance	Area
1	4.451	LiI (promoter)	2540358
2	9.182	AcOH (solv.)	7210865
3	12.884	dioxane (stand.)	70239
4	14.427	2-methylbutyric acid	390519
5	17.571	valeric acid	553632
6	20.799	2-methylvaleric acid	21259
7	26.506	hexanoic acid	26395

The flow rate of mobile phase was maintained at 1.0 mL/min.

Supplementary Table 24 The peak information in Supplementary Fig. 22.

Peak	Retention time [min]	Substance	Area
1	4.449	LiI (promoter)	1931919
2	9.389	AcOH (solv.)	13452942
3	12.917	dioxane (stand.)	59081
4	17.778	2-ethylbutyric acid	115790
5	20.706	2-methylvaleric acid	299831
6	26.133	hexanoic acid	451717

The flow rate of mobile phase was maintained at 1.0 mL/min.

Supplementary Table 25 The peak information in Supplementary Fig. 23.

Peak	Retention time [min]	Substance	Area
1	4.444	LiI (promoter)	2264668
2	9.496	AcOH (solv.)	13355094
3	12.875	dioxane (stand.)	63511
4	25.532	2-ethylvaleric acid	149371
5	30.314	2-methylhexanoic acid	302144
6	41.803	heptanoic acid	455350

The flow rate of mobile phase was maintained at 1.0 mL/min.

Supplementary Table 26 The peak information in Supplementary Fig. 24.

Peak	Retention time [min]	Substance	Area
1	8.872	LiI (promoter)	4141025
2	18.703	AcOH (solv.)	29661467
3	23.163	2-methylpropionic acid	246805
4	25.112	butyric acid	607646
5	28.498	2-methylbutyric acid	148381
6	31.070	DMSO (stand.)	169989
7	34.461	valeric acid	184935

The flow rate of mobile phase was maintained at 0.5 mL/min.

Supplementary Table 27 The peak information in Supplementary Fig. 25.

Peak	Retention time [min]	Substance	Area
1	4.450	LiI (promoter)	2087681
2	9.355	AcOH (solv.)	14736897
3	12.907	dioxane (stand.)	60275
4	14.455	2-methylbutyric acid	335933
5	17.638	valeric acid	409365
6	20.837	2-methylvaleric acid	14917
7	26.548	hexanoic acid	17504

The flow rate of mobile phase was maintained at 1.0 mL/min.

Supplementary Table 28 The peak information in Supplementary Fig. 26.

Peak	Retention time [min]	Substance	Area
1	4.438	LiI (promoter)	2005931
2	9.383	AcOH (solv.)	13482434
3	12.853	dioxane (stand.)	68813
4	20.513	cyclopentanecarboxylic acid	1046916

The flow rate of mobile phase was maintained at 1.0 mL/min.

Supplementary Table 29 The peak information in Supplementary Fig. 27.

Peak	Retention time [min]	Substance	Area
1	8.886	LiI (promoter)	4510300
2	18.105	AcOH (solv.)	4801815
3	20.951	propionic acid	348470
4	23.289	2-methylpropionic acid	39775
5	25.256	butyric acid	86687
6	28.666	2-methylbutyric acid	8585
7	31.124	DMSO (stand.)	205601
8	34.653	valeric acid	7775
9	59.805	phenol	2818193

The flow rate of mobile phase was maintained at 0.5 mL/min.

Supplementary Table 30 The peak information in Supplementary Fig. 28.

Peak	Retention time [min]	Substance	Area
1	8.874	LiI (promoter)	3521025
2	18.669	AcOH (solv.)	29622964
3	23.164	2-methylpropionic acid	154799
4	25.121	butyric acid	353941
5	28.500	2-methylbutyric acid	86956
6	31.079	DMSO (stand.)	139697
7	34.461	valeric acid	105460
8	59.642	phenol	2140101

The flow rate of mobile phase was maintained at 0.5 mL/min.

Supplementary Table 31 The peak information in Supplementary Fig. 29.

Peak	Retention time [min]	Substance	Area
1	4.457	LiI (promoter)	1784448
2	9.401	AcOH (solv.)	13348727
3	12.888	dioxane (stand.)	53744
4	14.471	2-methylbutyric acid	155388
5	17.620	valeric acid	220103
6	20.772	2-methylvaleric acid	10639
7	26.337	hexanoic acid	12567
8	30.948	phenol	1134231

The flow rate of mobile phase was maintained at 1.0 mL/min.

Supplementary Table 32 The peak information in Supplementary Fig. 30.

Peak	Retention time [min]	Substance	Area
1	4.456	LiI (promoter)	1750647
2	9.384	AcOH (solv.)	13503757
3	12.889	dioxane (stand.)	59876
4	14.472	2-methylbutyric acid	85168
5	17.653	valeric acid	113220

The flow rate of mobile phase was maintained at 1.0 mL/min.

Supplementary Table 33 The peak information in Supplementary Fig. 31.

Peak	Retention time [min]	Substance	Area
1	4.478	LiI (promoter)	2338351
2	9.493	AcOH (solv.)	14922681
3	12.891	dioxane (stand.)	81748
4	14.472	2-methylbutyric acid	126909
5	17.610	valeric acid	206384

The flow rate of mobile phase was maintained at 1.0 mL/min.

Supplementary Table 34 The peak information in Supplementary Fig. 32.

Peak	Retention time [min]	Substance	Area
1	4.452	LiI (promoter)	2168326
2	9.287	AcOH (solv.)	14988913
3	12.883	dioxane (stand.)	62228
4	14.470	2-methylbutyric acid	10583
5	17.677	valeric acid	37109

The flow rate of mobile phase was maintained at 1.0 mL/min.

Supplementary Table 35 The peak information in Supplementary Fig. 33.

Peak	Retention time [min]	Substance	Area
1	4.456	LiI (promoter)	2080262
2	9.325	AcOH (solv.)	14709383
3	12.899	dioxane (stand.)	77566
4	14.460	2-methylbutyric acid	150408
5	17.639	valeric acid	226000
6	20.866	2-methylvaleric acid	3335
7	26.453	hexanoic acid	17649
8	31.054	4-hydroxybenzoic acid	1320521

The flow rate of mobile phase was maintained at 1.0 mL/min.

Supplementary Methods

Preparation of Ir catalyst:

0.35 g $IrCl_4$ was dissolved in 25 mL methanol, the mixture was stirred vigorously at 70 ^oC for 2 h. Then $NaBH_4$ solution (0.76 g, dissolved in 25 mL water) was added slowly, followed by stirring for another 2 h. The as-synthesized Ir catalyst was obtained by centrifugation. The catalyst was washed with water and ethanol for 3 times respectively and dried under vacuum at 50 ^oC for 12 h.

Preparation of Ir/AC (Activated carbon supported Ir catalyst):

Ir/AC catalysts were prepared using wet chemical reduction procedure. Firstly, 0.35 g $IrCl_4$ was dissolved in 25 mL methanol, followed by adding 0.12 g activated carbon, after which the mixture was stirred vigorously at 70 ^oC for 8 h. Then $NaBH_4$ solution (0.76 g, dissolved in 25 mL water) was added slowly, followed by stirring for another 2 h. The product was collected by centrifugation, washed 3 times with water and then 3 times with ethanol, and dried under vacuum at 50 ^oC for 12 h.

Supplementary References

1. Haynes A. et al. Promotion of iridium-catalyzed methanol carbonylation: mechanistic studies of the Cativa process. *J. Am. Chem. Soc*. **126**, 2847-2861 (2004).
